# Hierarchical Multiscale Engineered Fe_3_O_4_/Ni Electrodes with Ultrafast Supercapacitive Energy Storage for Alternate Current Line‐Filtering

**DOI:** 10.1002/smsc.202200074

**Published:** 2022-12-14

**Authors:** Minjeong Kim, Byeong-Kwon Ju, Jin Gu Kang

**Affiliations:** ^1^ Nanophotonics Research Center Korea Institute of Science and Technology Seoul 02792 South Korea; ^2^ School of Electrical Engineering Korea University Seoul 02841 South Korea

**Keywords:** epitaxy, hierarchical multiscale engineering, nanosheets, periodic pores, ultrafast supercapacitors

## Abstract

Since the first demonstration of 3D bicontinuous porous architectures as a promising electrode for rechargeable batteries, many attempts have been made to extend this concept to other applications. Although some recent investigations have shown potential of bicontinuous structures as supercapacitor electrodes, there is a lack of capturing their capability for ultrafast charge/discharge as well as of manipulating the components in a more careful manner. Herein, novel bicontinuous porous Fe_3_O_4_/Ni supercapacitor electrodes fabricated by hierarchical multiscale engineering across three different length scales are reported. The electrodes comprise mesostructured epitaxial Ni scaffold (atomic‐scale), ultrathin pseudocapacitive Fe_3_O_4_ nanosheets (nanoscale), and interconnected periodic pores (mesoscale). It is highlighted that the electrodes can be cycled as a capacitor at an ultrahigh scan rate up to 100 V s^−1^ and also exhibit excellent line‐filtering properties at 120 Hz including the areal capacitance (272 μF cm^−2^), phase angle (−76°), and time constant (0.3 ms). This is attributed to the synergistic effects of rapid electron conduction, efficient utilization of surface‐limited reaction, and facile ion diffusion, enabled by engineering the properties at different levels of length scales. As a result, the electrode‐based symmetric supercapacitors enable remarkable line‐filtering performance with the significantly suppressed voltage ripple.

## Graphical abstract

Hierarchical multiscale engineering across the three different length scales (i.e., atomic‐scale, nanoscale, and mesoscale) is demonstrated as a new route to enabling ultrafast‐response supercapacitor electrodes. The engineered Fe_3_O_4_/Ni electrodes show excellent capacitive behaviors in response to ultrahigh voltage scan rate as well as 120 Hz alternating current switching.

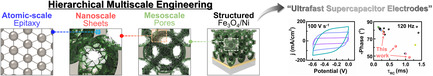

## 1 Introduction

Ultrafast response energy storage devices have become increasingly important for many technologies including rapid charging/discharging of electrified vehicles^[^ 1, 2 ^]^ and rectified alternate current (AC) line‐filtering.^[^ ^3^ ^]^ The criteria for “ultrafast response” here can be defined as the ability to charge/discharge devices within ≈10^−2^ s or to behave as a capacitor at high AC frequencies (>120 Hz).^[^ 4, 5, 6, 7, 8, 9, 10 ^]^ Supercapacitors, one class of electrochemical energy storage devices, are attractive for this purpose because, unlike diffusion‐limited lithium‐ion batteries, they store charge at the surface or subsurface of electrodes.^[^ 11, 12, 13, 14, 15, 16, 17 ^]^ Depending on the charge storage mechanism, supercapacitors are categorized into electrical double‐layer capacitors (EDLCs) and pseudocapacitors. For EDLCs, the charge is stored at the electrode surface via physisorption (non‐faradaic process) of electrolyte ions^[^ 11, 14, 15, 16, 18 ^]^; therefore, they would be suitable for an ultrafast response. One major drawback is, however, the low specific capacitance of carbonaceous materials used for EDLC electrodes.^[^ 12, 19, 20, 21, 22, 23 ^]^

Pseudocapacitors have received attention as an alternative route. Charge storage in pseudocapacitors is dominated by surface faradaic (i.e., redox) reactions, making them ideal for achieving both fast response and high capacitance.^[^ 11, 12, 13, 14, 24, 25 ^]^ Metal oxides such as RuO_2_, MnO_2_, V_2_O_5_, and spinel‐type oxides (e.g., Co_3_O_4_, Fe_3_O_4_) are representative materials that show pseudocapacitive behaviors.^[^ 3, 21, 26, 27, 28, 29, 30, 31, 32, 33, 34, 35, 36, 37, 38, 39, 40, 41, 42 ^]^ Despite their higher capacitances relative to EDLC electrodes, low electrical conductivities of most metal oxides, typically on the order of 10^−6^–10^−1^ S m^−1^,^[^ 23, 30, 43, 44 ^]^ limit their utility for ultrafast response applications. Exceptions exhibiting electrical conductivities close to those of half‐metals (≈10^4^ S m^−1^) or metals (**≈**10^6^ S m^−1^) include Fe_3_O_4_ and RuO_2_, with RuO_2_ receiving more attention.^[^ 45, 46, 47, 48 ^]^ Although RuO_2_ shows a large capacitance (800 F g^−1^) as well as capacitive behavior under ultrahigh scan rate (up to 1000 mV s^−1^), the biggest challenge is its high cost.^[^ ^49^ ^]^

Fe_3_O_4_, called magnetite, is another pseudocapacitive oxide with high electrical conductivity (≈10^4^ S m^−1^). It is well investigated that local hopping of electrons between Fe^2+^ and Fe^3+^ sites enables this half‐metallic conduction above Verwey transition temperature (≈120 K).^[^ 50, 51, 52, 53 ^]^ The additional benefits of Fe_3_O_4_ are its low cost, low toxicity, and natural abundance.^[^ ^54^ ^]^ As a result, there have been several studies on Fe_3_O_4_ as a supercapacitor electrode, most of which were oriented toward improving the capacitance by combining Fe_3_O_4_ with secondary materials such as carbon nanotubes, graphene, and reduced graphene oxides.^[^ 22, 55, 56, 57, 58, 59, 60, 61, 62, 63, 64, 65, 66, 67, 68, 69, 70, 71 ^]^ As far as we are aware, except for one study recently reported by our group,^[^ ^72^ ^]^ its potential as an ultrafast response electrode has never been highlighted so far. Our previous study successfully demonstrates that epitaxially grown 3D mesostructured Fe_3_O_4_ can respond as a pure capacitor to ultrafast changes in both DC and AC input. This is attributed to efficient electron and ion transport by high crystalline Fe_3_O_4_ matrix and 3D ordered macroporous structure, respectively.

While the approach based on 3D mesostructured epitaxy has proven effective, there are still more opportunities to improve electrochemical kinetics. As stated earlier, charge storage in pseudocapacitive oxides is primarily governed by surface‐limited redox reactions^[^ ^73^ ^]^; solid‐state diffusion barely contributes. It is, therefore, desirable to design the 3D mesostructure where the dimension of electrochemically active materials is at nanoscale.^[^ ^74^ ^]^ The pioneering works of Braun et al. in developing 3D bicontinuous mesostructured electrodes for rechargeable batteries are particularly important.^[^ ^75^ ^]^ They present ultrafast charging/discharge is possible when tens of nanometers‐thick electrochemically active materials are conformally coated on the 3D mesostructured metallic scaffolds. This is attributed to the bicontinuous architecture which is defined as a unique structure composed of a thin layer of electrochemically active materials sandwiched between the 3D interconnected electrolyte and conductive scaffold phases. Since the bicontinuous structure serves as a highly conductive pathway for electrons and a facile diffusion channel for ions in the liquid electrolyte, this is particularly attractive for pseudocapacitive oxides with low conductivity. As a result, there have been some attempts to extend this concept to pseudocapacitive oxides including NiO,^[^ ^76^ ^]^ MnO_2_,^[^ ^31^ ^]^ and Cu_2_O^[^ ^77^ ^]^; however, they failed to capture their capability for an ultrafast response. Furthermore, epitaxial engineering is lacking and electrochemically active materials are too thick in all these previous works.

**Figure 1:**
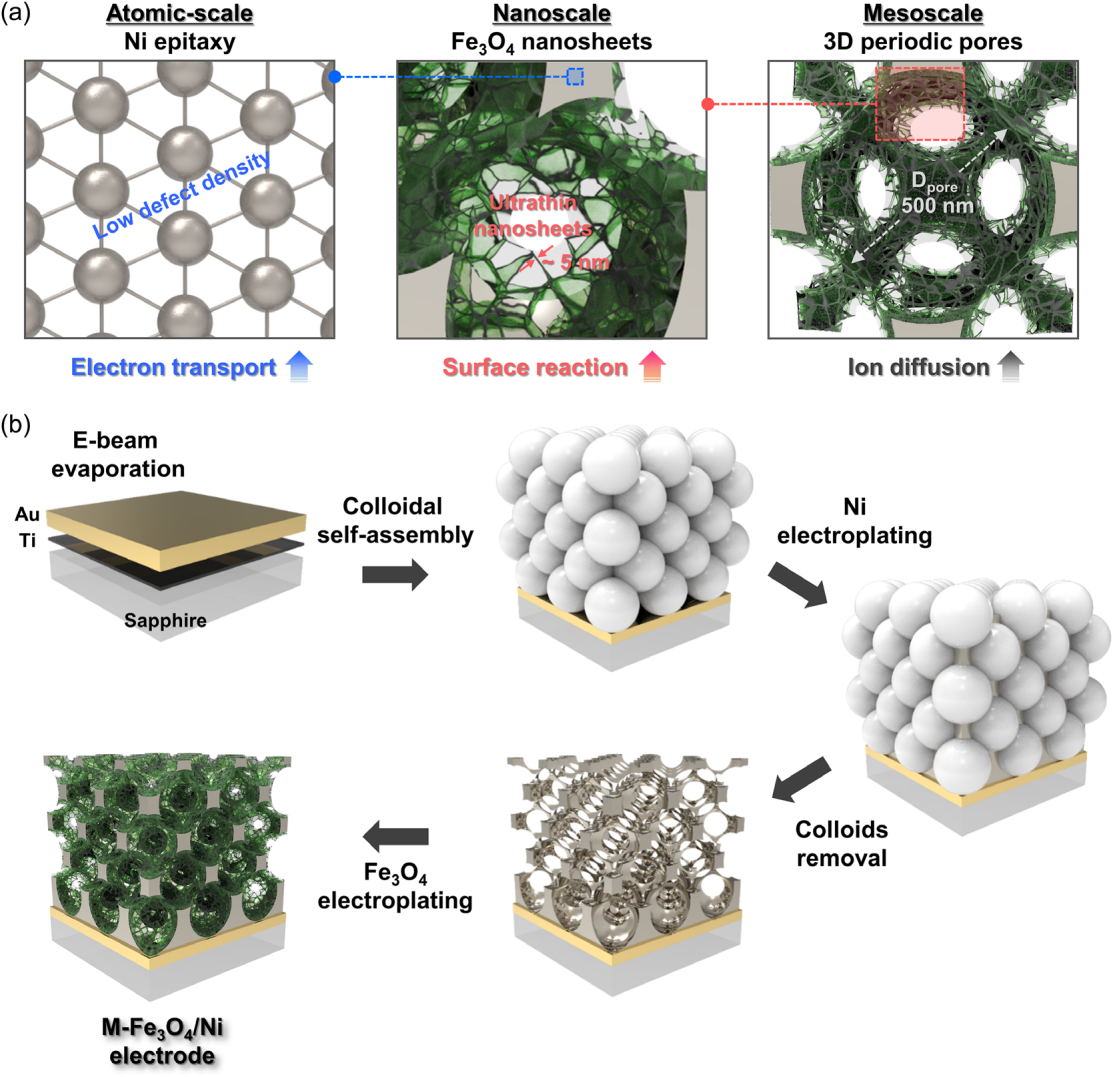
Schematic illustrations. a) Design principle and b) fabrication procedures of hierarchical multiscale engineered M‐Fe_3_O_4_/Ni supercapacitor electrodes.

Here, we demonstrate an advanced route to achieve ultrafast response charge storage by fabricating the hierarchical multiscale‐engineered Fe_3_O_4_/Ni electrode (M‐Fe_3_O_4_/Ni). As illustrated in **Figure** **1**a, we control factors affecting electrochemical reaction kinetics on all relevant length scales, including atomic‐scale electron transport, nanoscale surface reaction, and mesoscale ion diffusion. Specifically, the multiscale engineered electrode consists of pseudocapacitive Fe_3_O_4_ nanosheets vertically grown on a 3D periodic porous epitaxial Ni framework. The design principle of this electrode is to: 1) promote electron conduction through atomistic epitaxial Ni framework; 2) maximize the contribution of the surface‐limited (pseudo)capacitive process through Fe_3_O_4_ nanoscale sheet morphology; 3) facilitate electrolyte ion diffusion through the 3D mesostructure containing interconnected periodic pores. Benefitting from these, the M‐Fe_3_O_4_/Ni electrode can charge/discharge within 6 ms when cycled at 100 V s^−1^ and respond to AC line‐filtering frequency (120 Hz) with the excellent areal capacitance (272 μF cm^−2^), phase angle (−76°), and time constant (0.3 ms). When the symmetric two‐electrode supercapacitors based on the Fe_3_O_4_/Ni electrodes are used as the capacitor in the line‐filtering circuit, remarkable line‐filtering performance with the minimized voltage ripple is achieved. In addition, we also highlight our approach as a versatile platform for ultrafast storage applications by carefully regulating the number of M‐Fe_3_O_4_/Ni layers from one to three and five.

## 2 Results and Discussion

Figure 1b shows a fabrication schematic of the M‐Fe_3_O_4_/Ni electrode. A 100 nm‐thick epitaxial layer of Au (111) grown on a Ti (0001)/Al_2_O_3_ (0001) substrate is used as a seed layer in this work. It has been previously demonstrated that the relatively small lattice mismatches (Δ*a*) between layers (−2.3% for Au (111)/Ti (0001) and 3.0% for Ti (0001)/Al_2_O_3_ (0001)) enabled the multilayer heteroepitaxial growth.^[^ ^72^ ^]^ On the Au surface, 500 nm‐diameter polystyrene (PS) particles are self‐assembled to form periodic colloidal crystals. The voids inside the colloidal crystals are infiltrated with Ni by electrodeposition. The PS particles are removed by tetrahydrofuran (THF), leaving behind a 3D mesostructured Ni scaffold. The Fe_3_O_4_ nanosheets are uniformly grown on the Ni surface via pulsed electrodeposition, enabling the formation of M‐Fe_3_O_4_/Ni. As will be shown later, the 3D mesostructured Ni is epitaxial along both out‐of‐plane and in‐plane orientations (Δ*a* = −14% for Ni (111)/Au (111)).

**Figure 2:**
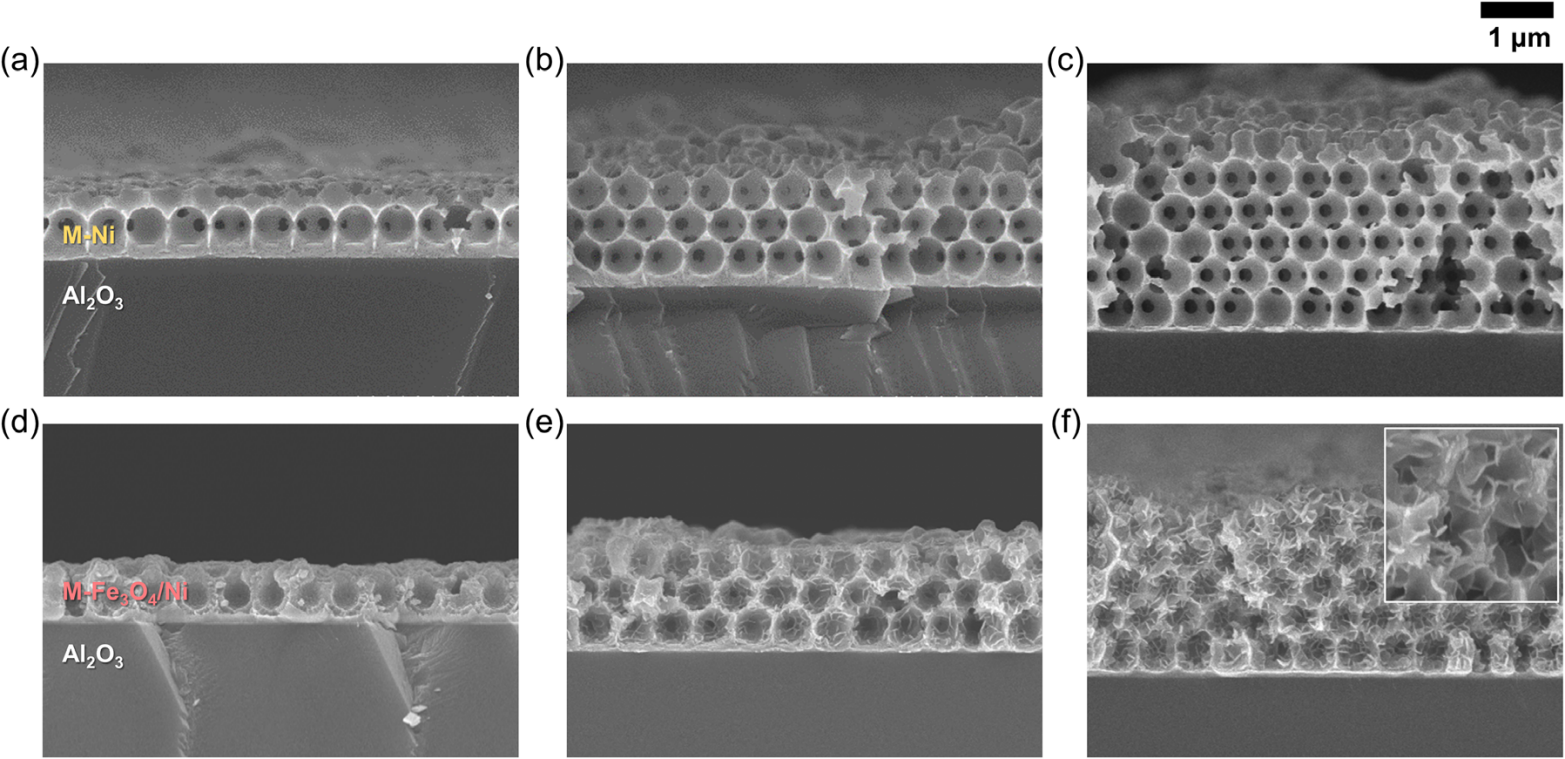
Morphology characterization. a–f) Cross‐sectional scanning electron microscopy (SEM) images of 3D mesostructured pristine Ni (M‐Ni) (a–c) and Fe_3_O_4_‐coated Ni (M‐Fe_3_O_4_/Ni) (d–f) with different numbers of porous layers: one (a,d), three (b,e), and five (c,f).

The cross‐sectional scanning electron microscopy (SEM) images of the samples are presented in **Figure** **2**. The number of porous Ni layers is controllable from one to three and five by adjusting the electrodeposition time (Figure 2a–c). Regardless of the number of layers, the formation of closed‐packed periodic pores is confirmed without showing any collapse of the Ni scaffolds, indicating successful selective removal of PS. We find that Fe_3_O_4_ layers are conformally deposited onto the curved Ni surfaces (Figure 2d–f), with the controlled amount of Fe_3_O_4_ deposited (Figure S1, Supporting Information). It is notable that the morphology of Fe_3_O_4_ varies depending on the number of porous Ni layers. The dense Fe_3_O_4_ film forms on one layer (Figure 2d), whereas the sheet‐like morphology becomes evident on the three and five layers (Figure 2e–f and inset). This is presumably because a sample with thicker porous Ni layers experiences larger ohmic polarization during pulsed electrodeposition, leading to a decrease in the magnitude of the effective cathodic pulses.

**Figure 3:**
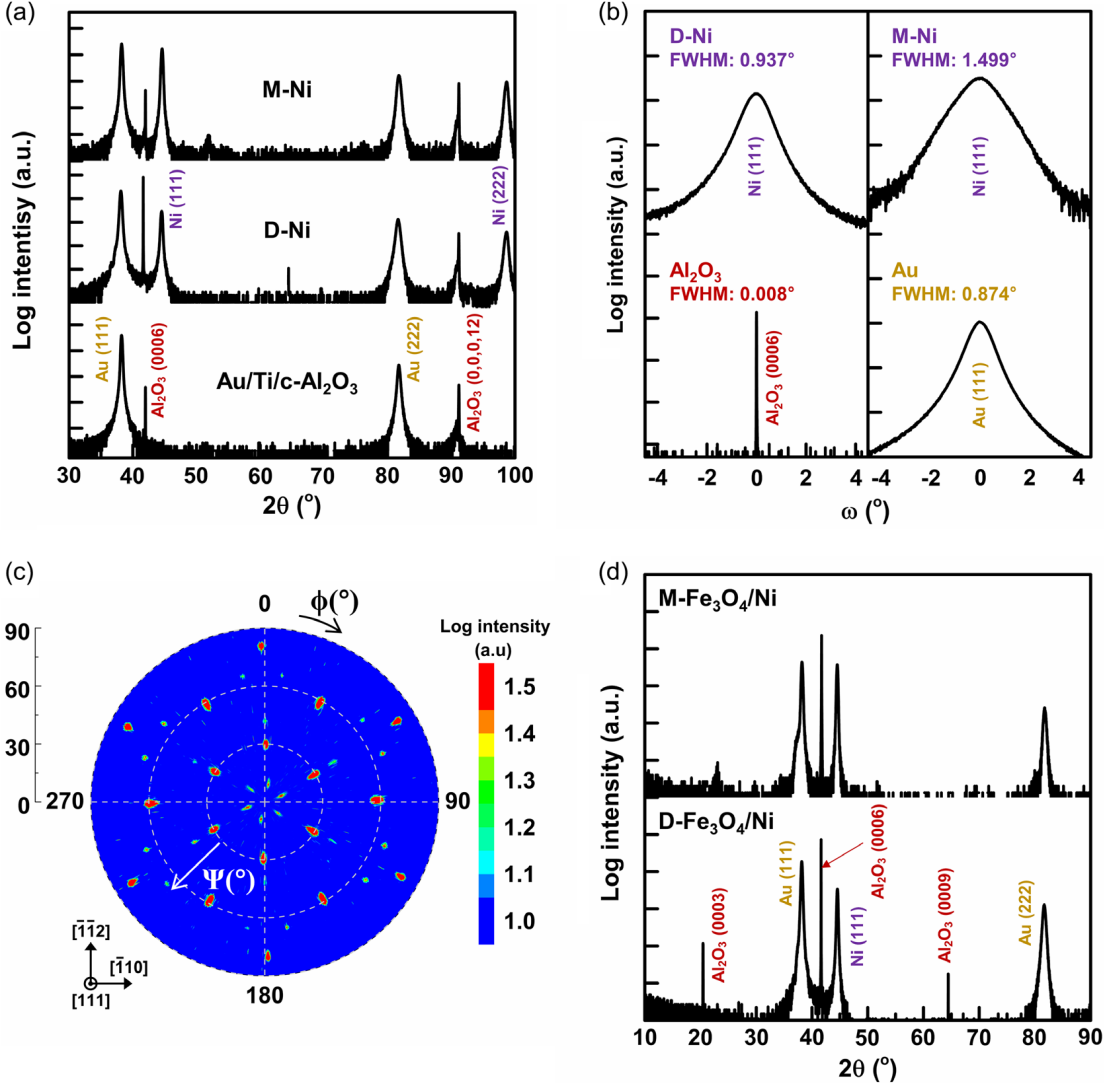
X‐ray diffraction (XRD). a) 2*θ*/*ω* scans of Au/Ti/c‐Al_2_O_3_ substrate (bottom), D‐Ni (middle), and M‐Ni (top) samples. b) Rocking curves of Al_2_O_3_ (0006) and Au (111) for Au–Ti/c–Al_2_O_3_ substrate (bottom). Rocking curves of Ni (111) for D‐Ni and M‐Ni (top). Full‐width half‐maximums (FWHMs) for each case are marked. c) Ni (311) pole figure for M‐Ni. *Ψ* and *ϕ* denote tilt and azimuth angles, respectively. d) 2*θ*/*ω* scans of D‐Fe_3_O_4_/Ni (bottom) and M‐Fe_3_O_4_/Ni (top).

Epitaxy of the deposited layers was investigated by X‐ray diffraction (XRD) as shown in **Figure** **3**. 2*θ*/*ω* measurement on the Au/Ti/c‐Al_2_O_3_ substrate (bottom of Figure 3a) shows the Au layer is epitaxial along an [111] out‐of‐plane orientation; the Ti (0002) reflection is very weak. Despite the relatively large lattice match (‐14%) between Ni (111) and Au (111), only Ni (111) and Ni (222) reflections are observed for both dense Ni (D‐Ni) (middle of Figure 3a) and 3D mesostructured Ni (M‐Ni) (top of Figure 3a). The crystallite sizes (*D*) of nickel were estimated with the Ni (111) peaks using the Scherrer equation,^[^ 72, 78 ^]^ yielding 31 nm for D‐Ni and 33 nm for M‐Ni, respectively. As calculated *D* values of D‐Ni and M‐Ni are similar, we expect no significant difference in matrix electrical conductivities. Rocking curves obtained from *ω* scans on the Ni (111) plane (Figure 3b) exhibit the full‐width half‐maximum (FWHM) of M‐Ni, which is ≈1.6 times larger than that of D‐Ni, representing M‐Ni that has a larger mosaic spread than D‐Ni. This agrees with the previous observation that the presence of colloidal templates during epitaxial growth leads to structural imperfections.^[^ ^79^ ^]^

The Ni (311) pole figure of M‐Ni in Figure 3c shows discrete {311} reflections at *Ψ* = 30, 58, and 80°, indicating high‐quality epitaxy along the in‐plane orientation. The six reflections separated azimuthally (*φ*) by 60° at *Ψ* = 30 and 80° indicate two Ni {111} domains that are antiparallel to each other.^[^ ^80^ ^]^ When deposited on the c‐Al_2_O_3_, Au (111) and Ti (0001) layers are also known to consist of two domains 180° rotated to each other.^[^ 53, 81 ^]^ Therefore, two types of in‐plane epitaxial relationships with respect to the sapphire are present in M‐Ni: Ni (111)$\left[1\bar{1}0\right]$//Au (111)$\left[1\bar{1}0\right]$//Ti (0002)$\left[1\bar{2}10\right]$//Al_2_O_3_ (0006)$\left[1\bar{1}00\right]$ (parallel) and Ni (111)$\left[\bar{1}10\right]$//Au (111)$\left[\bar{1}10\right]$//Ti (0002)$\left[\bar{1}2\bar{1}0\right]$//Al_2_O_3_ (0006)$\left[1\bar{1}00\right]$ (antiparallel). The six weak reflections at *Ψ* = 9° result from a small number of Ni {511} mirror twins made of six domains.^[^ ^53^ ^]^

A 2*θ*/*ω* scan on the M‐Fe_3_O_4_/Ni sample shows no peaks other than those of Al_2_O_3_, Au, and Ni (top of Figure 3d), indicating amorphous or poorly crystalline Fe_3_O_4_ forms. This behavior is also observed when Fe_3_O_4_ was deposited on D‐Ni (bottom of Figure 3d). We attribute this to insufficient time for the nuclei to crystallize during pulsed potentiostatic electrodeposition. Figure S2, Supporting Information, supports our interpretation. The epitaxial Fe_3_O_4_ (111) film forms on the Ni (111) surface by continuous potentiostatic electrodeposition.

**Figure 4:**
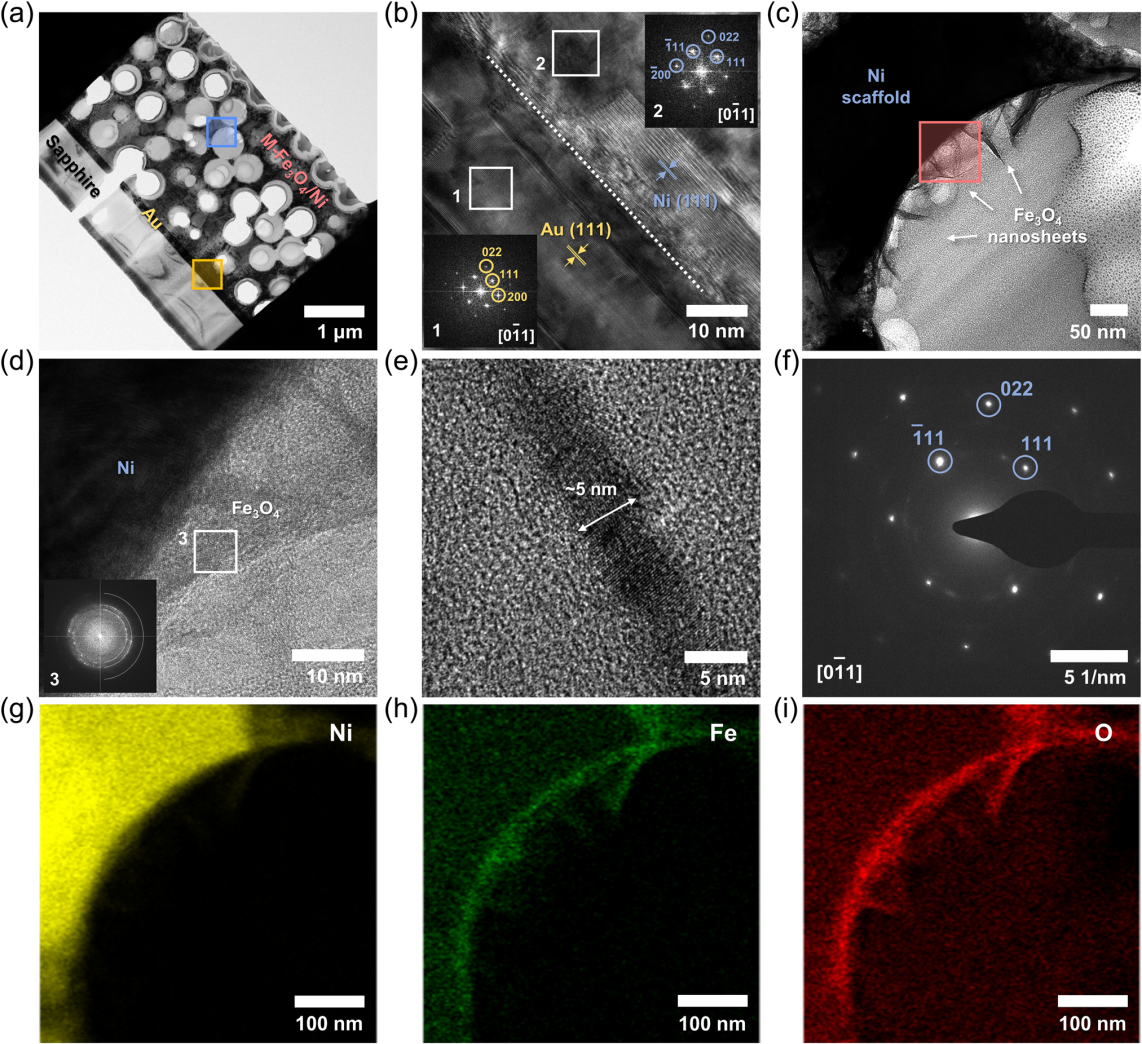
Microstructure characterization of M‐Fe_3_O_4_/Ni. a) Cross‐sectional low‐magnification transmission electron microscopy (TEM) image of the sample milled by focused ion beam (FIB). b,c) Magnified views of orange square (b) and blue square (c) in (a). Insets of (b) are fast Fourier transform (FFT) patterns acquired at two white squares. d) Magnified view of red square in (c) and FFT pattern (inset) of the white square. e) High‐resolution TEM (HRTEM) image of a single nanosheet with ≈5 nm in thickness. f) Selected‐area electron diffraction (SAED) pattern acquired from (c). g–i) Energy‐dispersive X‐ray spectroscopy (EDX) elemental mapping of (c) for Ni (g), Fe (h), and O (i).

To gain a more complete understanding of the microstructure and crystallography, transmission electron microscopy (TEM) analysis was performed on the cross‐section of an M‐Fe_3_O_4_/Ni sample milled by a focused ion beam (FIB) as shown in **Figure** **4**a. A high‐resolution TEM (HRTEM) image (Figure 4b) taken from the interface between the Ni and Au layers (orange square) exhibits that both layers are oriented along the [111] direction; for each case, the measured distance between two atomic planes corresponds to the *d*‐spacing in the JCPDS reference (2.03 Å for Ni (111) and 2.35 Å for Au (111)). For the Au layer, the fast Fourier transform (FFT) pattern (inset 1 of Figure 4b) is indexed to the reflections of the atomic planes that appear when viewed along the zone axis, indicating it is single crystalline. Most regions of the Ni layer exhibit a similar pattern (inset 2 of Figure 4b) signifying the single crystal, but some local regions contain defects including twins and stacking faults. This may be because of the relatively large lattice mismatch between Ni and Au.

Figure 4c presents a magnified TEM image of the blue square in Figure 4a. The curved Ni surface is decorated with randomly oriented, entangled Fe_3_O_4_ nanosheets with ≈100 nm in length. This characteristic is more evident in Figure 4d, a zoom‐in image of the red square in Figure 4c. The FFT pattern collected from overlapped nanosheets (inset of Figure 4d) exhibits a few non‐periodic weak reflections, indicating poor crystallinity. The HRTEM image of a single nanosheet (≈5 nm in thickness) in Figure 4e supports this. The periodic arrangement of atomic planes is barely observed in the nanosheet, which is in significant contrast to the Ni and Au layers. Figure 4f shows a selected area electron diffraction (SAED) pattern acquired over the area of 1 × 1 μm^2^. The diffraction spots are indexed with a $\left[0\bar{1}1\right]$ zone axis of Ni. This once again demonstrates that M‐Fe_3_O_4_/Ni is comprised of the porous epitaxial Ni scaffold coated with the poorly crystalline Fe_3_O_4_ phase. The elemental mappings by energy‐dispersive X‐ray spectroscopy (EDX) (Figure 4g–i) indicate that pulse electrodeposition enables uniform coating of Fe_3_O_4_ layers on the Ni surface.

**Figure 5:**
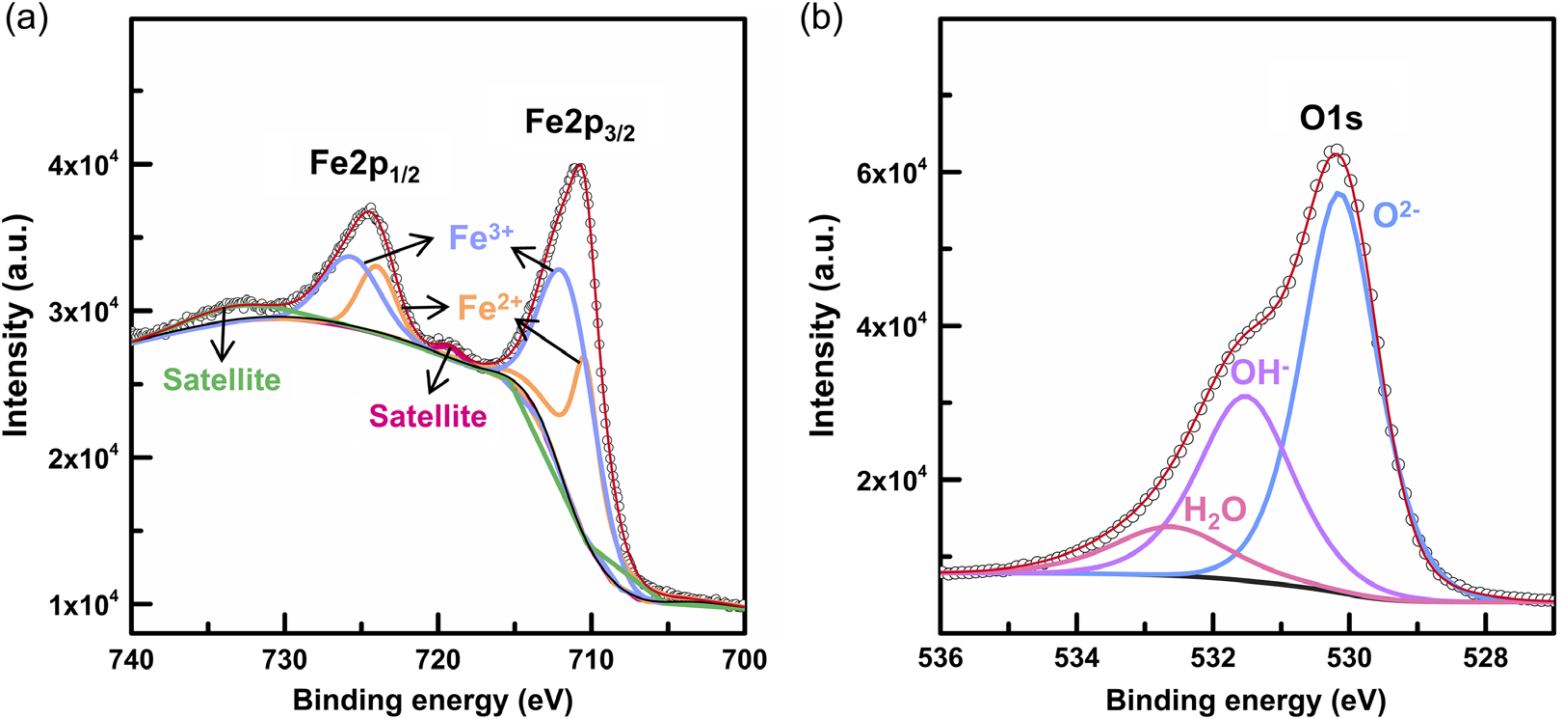
Chemical state analysis. a,b) High‐resolution XPS spectra of Fe 2p (a) and O 1s (b).

To identify the exact composition of the nanosheets, we collected the X‐ray photoelectron spectroscopy (XPS) spectra of M‐Fe_3_O_4_/Ni as shown in Figure S3, Supporting Information (survey) and **Figure** **5** (high‐resolution narrow). In the narrow Fe 2p spectrum over the binding energy range of 700–740 eV (Figure 5a), two peaks corresponding to Fe 2p_1/2_ (centered at 724.5 eV) and Fe 2p_3/2_ (710.9 eV) are observed. Each peak was fitted with a convoluted form comprising two curves of Fe^2+^ and Fe^3+^ oxidation states with a goodness‐of‐fit of ≈0.998. For both Fe 2p_1/2_ and Fe 2p_3/2_ cases, the ratio between the integrated intensities of Fe^2+^ and Fe^3+^ curves ([Fe^2+^]/[Fe^3+^]) is 0.65, indicating the nonstoichiometry (*δ*) of Fe_3−δ_O_4_ is −0.07.^[^ ^82^ ^]^ As our sample is at a lower oxidation state (Fe‐excess) than stoichiometric Fe_3_O_4_ (*δ* = 0), we expect it to be an n‐type semiconductor with good electrical conduction enabled by atomic‐scale hopping of electrons between Fe^2+^ and Fe^3+^ sites.^[^ ^83^ ^]^ The narrow O1s spectrum in Figure 5b can be fitted by the convolution of three curves: O^2−^, OH^−^, and H_2_O. The presence of OH functional groups renders the sample surface hydrophilic, thereby improving its ability to wet the capacitor electrolyte. The Raman spectrum (Figure S4, Supporting Information) exhibits three peaks at the Raman shifts of 306 (*E*_g_), 539 (*T*_2g_(2)), and 667 cm^−1^ (*A*_1g_), a typical characteristic of the Fe_3_O_4_ phase.^[^ ^84^ ^]^ The first two peaks are weak and broad, implying poor crystallinity.

**Figure 6:**
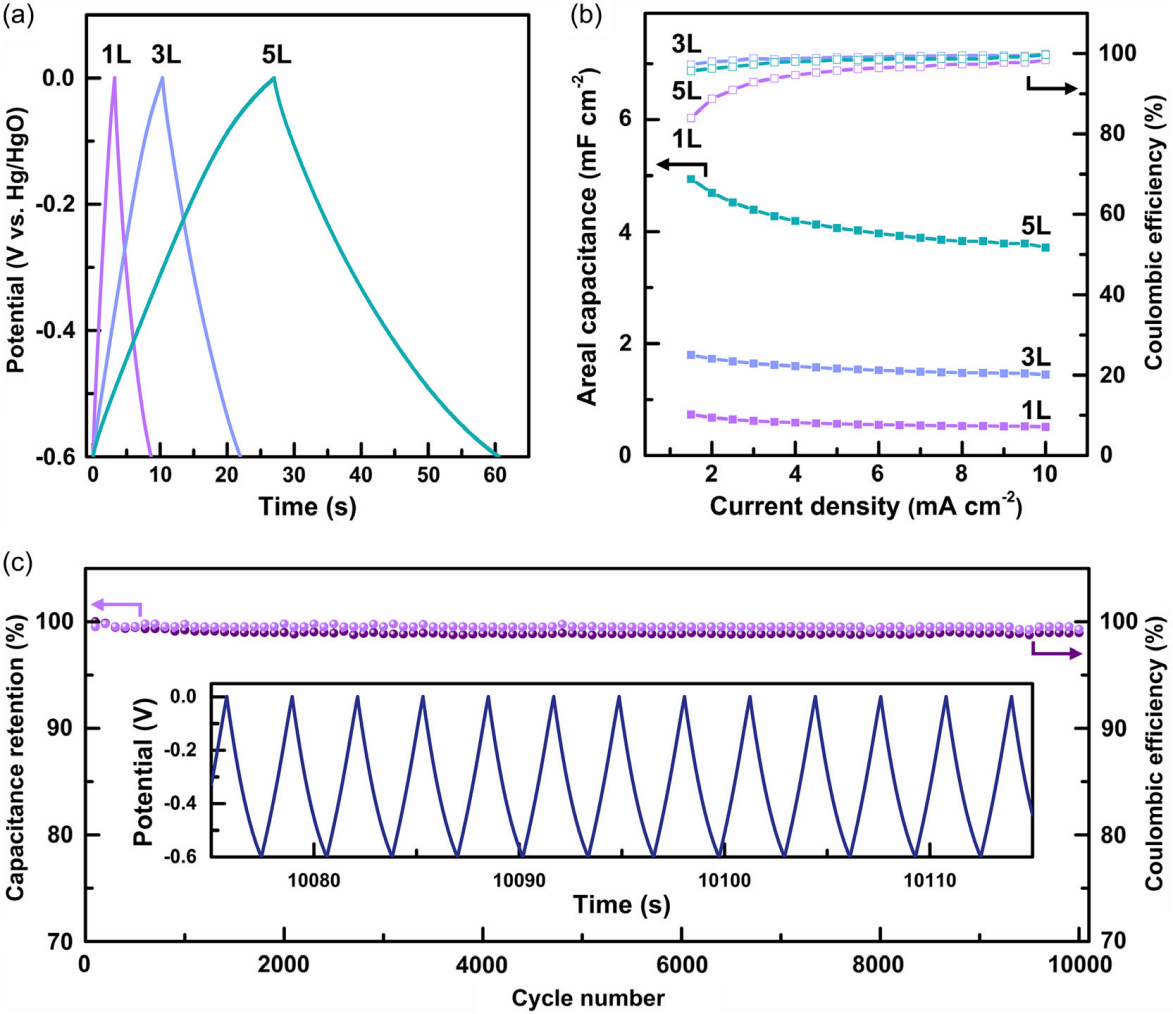
Galvanostatic charge–discharge (GCD) tests. a) Potential profiles at 1.5 mA cm^−2^ between −0.6 and 0 V (vs Hg/HgO) for 1L, 3L, and 5L. b) Areal capacitance (left axis) and Coulombic efficiency (C.E.; right axis) as a function of current density applied. c) Capacitance retention (left axis) and C.E. (right axis) over 10 000 cycles for 3L. Inset is its corresponding GCD profile at 10 mA cm^−2^.

Since the thickness of the M‐Fe_3_O_4_/Ni electrode can be precisely controlled as demonstrated earlier, we expect this to offer a versatile platform applicable in ultrafast energy storage. To test this idea, we compare the electrochemical properties of three types of M‐Fe_3_O_4_/Ni electrodes with different numbers of porous layers: one (1L), three (3L), and five (5L). **Figure** **6**a shows the galvanostatic charge–discharge (GCD) voltage profiles between −0.6 and 0 V (vs Hg/HgO) at the current density of 1.5 mA cm^−2^ for the three electrodes. The charge–discharge curves exhibit an isosceles triangle with slight distortion, implying that charge storage is dominated by pseudocapacitance.^[^ 73, 85 ^]^ As far as we are aware, the origin of Fe_3_O_4_ pseudocapacitance in an alkaline aqueous medium has not yet been clearly identified. However, we describe the most likely mechanism for Fe_3_O_4_ pseudocapacitance in Note S1, Supporting Information. With increasing current density up to 10 mA cm^−2^, the shape of curves becomes a nearly perfect triangle (Figure S5, Supporting Information), a sign of EDLC as a predominant process.^[^ 12, 73, 86, 87 ^]^ It is notable that ohmic voltage drops (IR) that typically appear at the beginning of charging and discharging due to the internal resistances are negligible. We attribute this to excellent electron transport enabled by the high electrical conductivity of the single crystalline Ni scaffolds.^[^ ^88^ ^]^ Figure 6b exhibits the rate‐dependent areal capacitances and the corresponding Coulombic efficiencies. Over the entire range of the current density (1.5–10 mA cm^−2^), 5L shows the largest capacitances, followed by 3L and 1L. This can be attributed to the larger surface area of 5L which affords more sites accessible for the electrolyte ions. The dependence of the surface area on the number of porous layers is mathematically calculated using the SEM images and the packing density of FCC‐type colloidal crystals (see Figure S6 and Note S2, Supporting Information). At the highest current density applied (10 mA cm^−2^), 5L still retains 75% of its lowest current (1.5 mA cm^−2^) capacitance, representing its good rate‐retention ability. This once again confirms that M‐Fe_3_O_4_/Ni can serve efficient and rapid pathways for ion and electron delivery even when the geometry is quite complex (i.e., 5L).

Pseudocapacitive electrodes are typically known to exhibit poor cycling properties due to the degradation of the internal structure and thus electrical disconnection of active materials during cycling.^[^ ^89^ ^]^ However, we find our sample (3L) demonstrates exceptional cycling stability, as presented in Figure 6c. The electrode delivers ≈99% of its initial capacitance and retains ≈99% Coulombic efficiency over 10 000 cycles at 10 mA cm^−2^. This is rather surprising because Fe_3_O_4_ nanosheets underwent structural collapse after cycling due to the surface redox reactions (Figure S7, Supporting Information). The primary reason for good capacity retention is that the 3D architectured Ni scaffold responsible for electrical connectivity was preserved. Another reason is that electrical conduction in Fe_3_O_4_ was enhanced at the expense of the decreased surface area of collapsed structure; charge is carried more effectively in the collapsed structure perhaps with isotropy relative to the anisotropic nanosheets.

**Figure 7:**
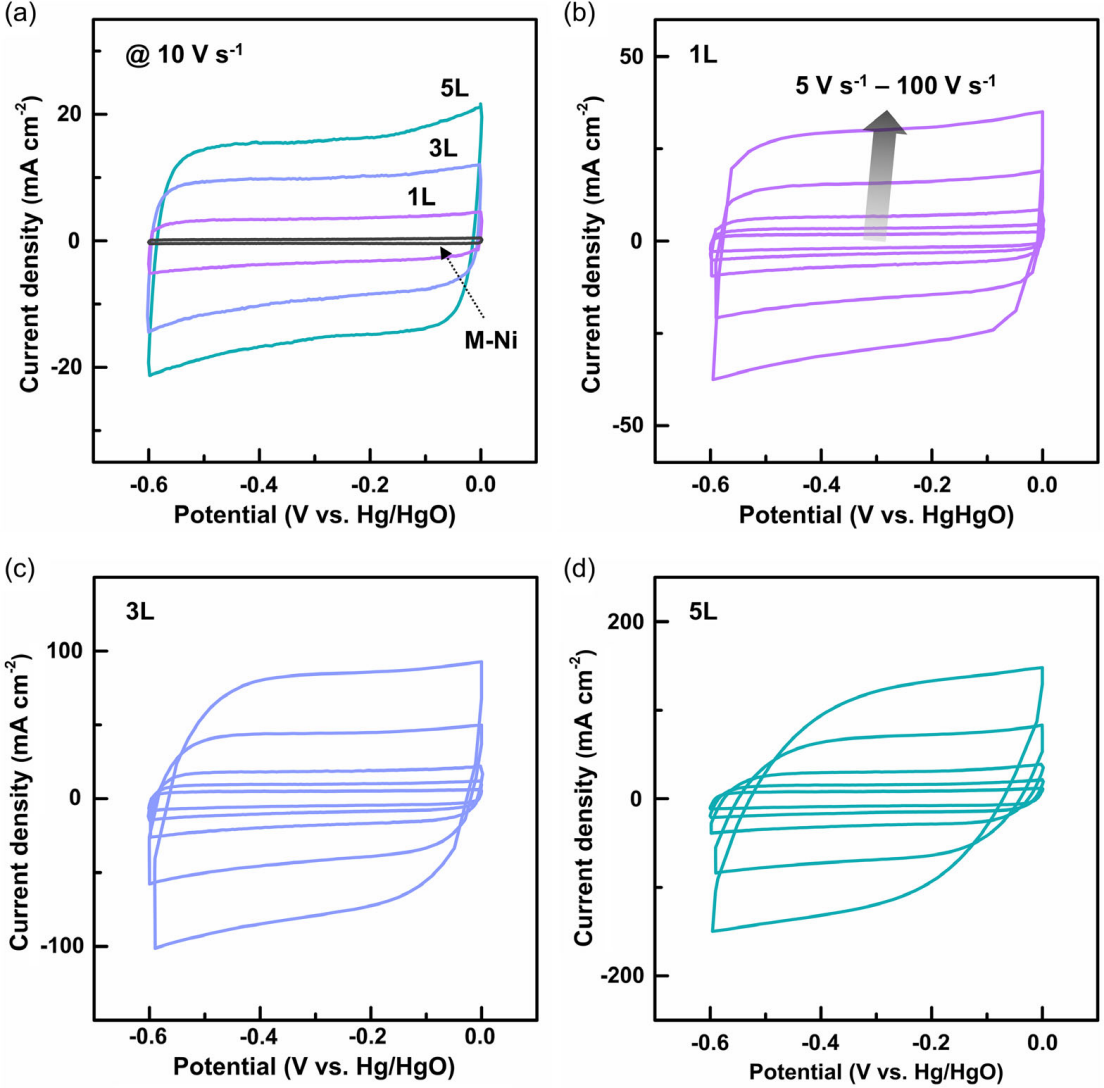
Ultrahigh scan‐rate response. a) Cyclic voltammetry (CV) curves at 10 V s^−1^ for three types of M‐Fe_3_O_4_/Ni electrodes (1L, 3L, 5L) and M‐Ni as a control. b–d) CV curves at scan rates ranging from 5 to 100 V s^−1^ for 1L (b), 3L (c), and 5L (d).

One of the most significant results in this study is that the multiscale engineered electrodes are able to behave as a capacitor in response to ultrafast voltage scan. **Figure** **7**a shows the cyclic voltammetry (CV) curves collected between −0.6 and 0 V (vs Hg/HgO) at a rate of 10 V s^−1^. The negligible curve area for mesostructured epitaxial Ni (M‐Ni) provides evidence for its little contribution to the capacitance. All M‐Fe_3_O_4_/Ni electrodes retain a nearly rectangular shape with symmetric anodic/cathodic currents, demonstrating their ability to store energy even at a very high scan rate. Perhaps surprisingly, we find that this characteristic is stable over repeated charge/discharge cycles and is maintained with small distortion in the curve shape up to an ultrahigh rate of 100 V s^−1^, regardless of the number of porous layers (Figure 7b–d). In other words, the capacitances of 0.27 (1L), 0.64 (3L), and 0.86 mF cm^−2^ (5L) (computed using Equation (S1), Supporting Information) can be delivered within as short as 6 ms when cycled at 100 V s^−1^, corresponding to ultrafast charge and discharge. As far as we are aware, such performance has not yet been demonstrated so far from any electrode materials except for carbon‐based materials,^[^ 6, 7, 8, 9, 10, 90, 91, 92, 93, 94, 95, 96, 97, 98, 99, 100 ^]^ RuO_2_,^[^ ^101^ ^]^ transition metal chalcogenides,^[^ 102, 103 ^]^ TiN,^[^ ^104^ ^]^ and MXene;^[^ ^105^ ^]^ however, these materials are costly or require complex fabrication processes. This highlights the power of our approach (i.e., hierarchical multiscale engineering) as a promising tool for boosting electrochemical charge delivery. Based on the analysis^[^ ^12^ ^]^ (see Note S3, Supporting Information) of the CV curves acquired at varying scan rates ranging from 10 mV s^−1^ to 100 V s^−1^ (Figure S8a–c, Supporting Information), we reveal that for all M‐Fe_3_O_4_/Ni electrodes, EDLC becomes increasingly dominant over pseudocapacitance as the scan rate goes beyond 1 V s^−1^ (Figure S8d–i, Supporting Information). This could be another factor enabling ultrafast energy storage.

**Figure 8:**
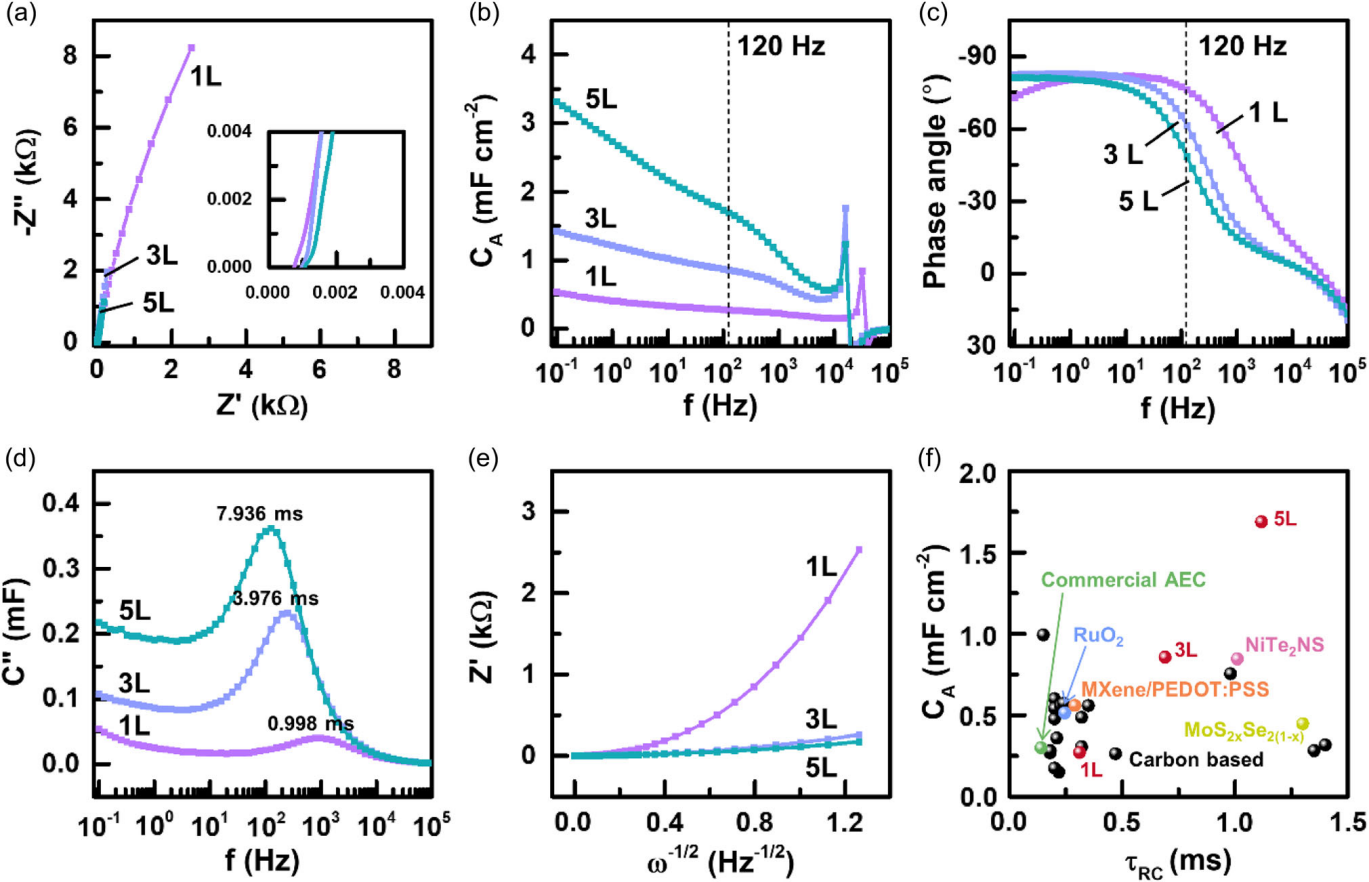
Electrochemical impedance spectroscopy (EIS) of M‐Fe_3_O_4_/Ni. a) Nyquist plots over the entire frequency range. The inset shows the magnified plots at a high‐frequency range. b) Areal capacitance (*C*_A_) versus frequency. c) Phase angle versus frequency. d) Imaginary part of the complex capacitance (*C′*) versus frequency. e) Randles plots (*Z'* vs *ω* ^−1/2^). f) A summarized graph plotting *C*_A_ vs *τ*_RC_ for M‐Fe_3_O_4_/Ni electrodes (red circles) and other previously reported electrodes for comparison.

Materials that function as a capacitor under an ultrafast scan speed have proven effective for line‐filtering the rectified alternating current (AC) voltage, referred to as AC line‐filtering.^[^ ^106^ ^]^ As it is the process of smoothening AC voltage ripples at second‐harmonic (or higher‐order‐harmonic) frequency of the global standard frequency (typically 50–60 Hz), three properties at 120 Hz including the areal capacitance (*C*_A_), phase angle, and resistor–capacitor (RC) time constant (*τ*_RC_) are of importance.^[^ ^107^ ^]^ To evaluate these, we carried out electrochemical impedance spectroscopy (EIS) measurements over the frequency range of 2 MHz to 0.1 Hz. **Figure** **8**a presents the Nyquist plots for 1L, 3L, and 5L. The equivalent series resistances estimated from the intercept of the real axis (*Z'*) are very small for all cases: 0.028 for 1L, 0.088 for 3L, and 0.126 Ω for 5L, respectively. This again confirms excellent electrical conduction and ion migration through 3D periodic porous structures containing highly crystalline metallic scaffold. At the high‐frequency range (inset of Figure 8a), the semicircle is not observed for any electrodes, indicating significantly efficient charge transfer at the electrode/electrolyte interfaces.^[^ ^108^ ^]^ A nearly vertical line in the low‐frequency regions represents that the response of the electrodes is purely capacitive at low frequencies.^[^ ^109^ ^]^ Frequency‐dependent responses of the areal capacitance (*C*_A_) calculated by Equation (S2), Supporting Information, are plotted in Figure 8b. Up to 10 kHz, all M‐Fe_3_O_4_/Ni electrodes retain capacitive characteristics, proving their capability to accumulate/release charges at a very high switching speed. At 120 Hz, *C*_A_ are 272, 856, and 1688 μF cm^−2^ for 1L, 3L, and 5L, respectively; *C*_A_ scales approximately linearly with the number of porous layers. However, the *C*_A_ ratio (6.2) between 5L and 1L is a factor of ≈1.24 (= 6.2/5) larger than the surface area ratio (5). We attribute this to the Fe_3_O_4_ morphology difference between the two samples. As stated earlier, 1L contains the packed Fe_3_O_4_ film whereas 5L contains the nanosheets. The surface area of the nanosheets is larger relative to the packed state, yielding a slightly larger *C*_A_ than expected for 5L. *C*_A_ at 120 Hz is of importance because the amplitude of the voltage ripple is inversely proportional to *C*_A_^[^ 110, 111 ^]^ We note that *C*_A_ for 5L (1688 μF cm^−2^) is 5.6 times larger relative to the commercial aluminum electrolytic capacitor (AEC; 300 μF cm^−2^) and is equivalent to or exceeds those of many electrodes to date achieved with other methods^[^ 5, 6, 7, 8, 9, 10, 90, 91, 92, 93, 94, 95, 96, 97, 98, 99, 100, 101, 102, 103, 104, 105, 106, 112, 113, 114, 115, 116, 117, 118 ^]^ (Table S1, Supporting Information). This can be attributed to the contribution of the nearly entire portion of active materials (i.e., Fe_3_O_4_ nanosheets) to (sub)‐surface‐limited charge storage due to their ultrathin feature.

The phase angle at 120 Hz is another critical parameter affecting AC line‐filtering performance; a value close to −90° corresponds to an ideal capacitor.^[^ ^119^ ^]^ Figure 8c presents the Bode plots of the phase angle (Equation (S3), Supporting Information) as a function of the frequency. The phase angle at 120 Hz becomes larger as the number of porous layers increases: −76.2° (1L), −61.3° (3L), and −48.5° (5L). This can be attributed to the increase in the internal resistance and thus *Z'* with an increasing number of porous layers of the electrode. It is notable that 1L exhibits a phase angle comparable to those of the AEC (−83.9°) and many other carbon‐based electrodes^[^ 5, 6, 7, 8, 10, 91, 94, 96, 97, 99, 106, 114, 115 ^]^ (Table S1, Supporting Information). Even for 3L and 5L, the values are equal to or smaller than those attained by other non‐carbonaceous materials.^[^ 102, 103, 104, 118 ^]^ Along with *C*_A_ and the phase angle, *τ*_RC_ plays an essential role in determining the quality of the line‐filtered signal. The computed *τ*_RC_ at 120 Hz (Equation (S4), Supporting Information) for 1L, 3L, and 5L are 0.31, 0.69, and 1.12 ms, respectively (Table S1, Supporting Information), showing a trend the same as those for *C*_A_ and the phase angle. While *τ*_RC_ for our M‐Fe_3_O_4_/Ni electrodes are 2.2, 4.9, and 8 times longer relative to the AEC (0.14 ms), they are sufficiently shorter than the upper bound to the time period (*τ*_max_ = 8.3 ms) required for line‐filtering. Not being directly related to line‐filtering, the relaxation time constant (*τ*_0_)—the minimum time required to discharge all the energy at the efficiency over 50%^[^ 86, 120 ^]^—is often used to examine the response kinetics of supercapacitor electrodes. The frequency where the imaginary part (*C′*) of the complex capacitance (Equation (S5), Supporting Information) peaks is equal to 1/*τ*_0_ (Figure 8d). *τ*_0_ of three electrodes (1.0 for 1L, 4.0 for 3L, and 7.9 ms for 5L) fall below 10^−2^ s, demonstrating their ability to charge/discharge at ultrahigh rates.

It is necessary to point out that the increase in *τ*_RC_ (3.6 times) is not as much as the increase in *C*_A_ (6.2 times) as the number of layers increases from one to five. Since *τ*_RC_ can be expressed as *Z'*AC_A_ based on the relation between Equation (S2) and (S4), Supporting Information, the only factor that yields the increase in *τ*_RC_ smaller than the increase in *C*_A_ by a factor of 1.7 (≈6.2/3.6) is the difference in *Z'*; the electrode areas (*A*) are the same for all samples. *Z'* at 120 Hz is determined by the combined effects of ion migration, electrical conduction, and ion diffusion.^[^ ^121^ ^]^ Due to the sufficiently high electrolyte concentration (6 m KOH), ion migration is presumably independent of the number of layers. The electrical resistance (the reciprocal of the electrical conductance) of 5L is 5 times higher relative to 1L because of a factor of 5 greater thickness of 5L; all M‐Fe_3_O_4_/Ni exhibit the same effective electrical conductivity. Therefore, we expect that ion diffusion through 5L is much faster compared to 1L. To validate this, we estimated the chemical diffusion coefficients of ions (*D*_*i*_) using the Randles plots in Figure 8e. The slope at long time scales gives the Warburg impedance (*k*_w_) which is inversely proportional to the square root of *D*_*i*_ as illustrated in Equation (S6), Supporting Information. Perhaps surprisingly, we find *D*_*i*_ of 5L (6.8 × 10^−8^ cm^2^ s^−1^) is approximately two orders of magnitude greater than that of 1L (5.6 × 10^−10^ cm^2^ s^−1^), in good agreement with our expectation. Our qualitative interpretation of faster diffusion in 5L relative to 1L is provided in Note S4 and Figure S9, Supporting Information.

While 1L exhibits the smallest areal capacitance (*C*_A_) among three M‐Fe_3_O_4_/Ni electrodes, it is only a factor of 1.1 smaller relative to the commercial AEC; the other two electrodes show significantly larger *C*_A._ Therefore, we focus our attention on *C*_A_ and *τ*_RC_ as a figure‐of‐merit for evaluating line‐filtering performance. Figure 8f presents a plot of *C*_A_ versus *τ*_RC_ for our electrodes and those previously reported elsewhere.^[^ 5, 6, 7, 8, 9, 10, 90, 91, 92, 94, 96, 97, 98, 99, 100, 101, 102, 105, 106, 113, 114, 115, 118 ^]^ It is clear that 1L demonstrates the excellent *τ*_RC_ which falls within the range of those achieved by many carbonaceous electrodes^[^ 10, 92, 97, 98, 100, 114, 115 ^]^ and are slightly poorer relative to the AEC. Even for 5L, *τ*_RC_ is close to those of NiTe_2_ nanosheets^[^ ^118^ ^]^ which have proven effective for line‐filtering. Additionally, *C*_A_ of 5L surprisingly beats those of supercapacitor electrodes ever reported. This highlights the superiority of our 5L electrodes. A summarized plot of the phase angle against *τ*_RC_ for our electrodes and the previously reported electrodes is also depicted in Figure S10, Supporting Information for reference; 1L shows the phase angle that is comparable to those of most carbonaceous electrodes. Overall, hierarchical multiscale engineering offers routes to designing various types of ultrafast capacitor electrodes, ranging from that (5L) with large *C*_A_ and small *τ*_RC_ to that (1L) with a small phase angle and small *τ*_RC_.

**Figure 9:**
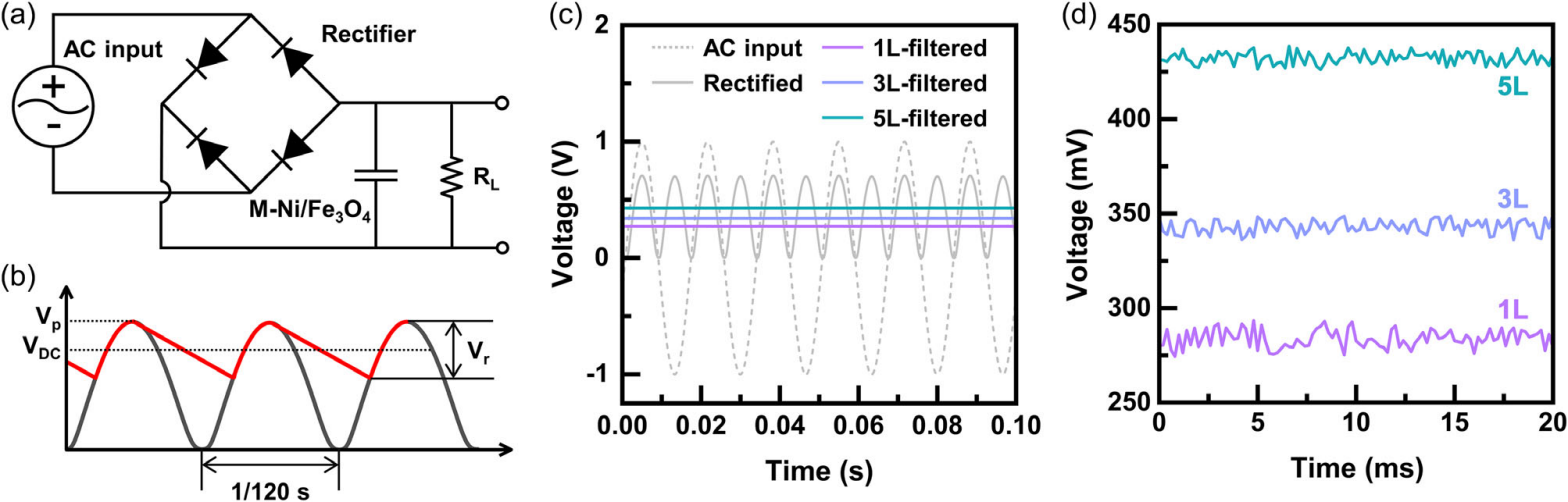
AC line‐filtering. a) Schematic of the electric circuit of AC line‐filtering experiment. b) A graph illustrating that the rectified 120 Hz signal with the maximum amplitude *V*_p_ (solid black line) is smoothened to the line‐filtered signal (solid red line) with the mean voltage value of *V*_DC_ and the leftover ripple of *V*_r_. c) A plot of voltage versus time for the 60 Hz AC input (dashed grey line), the 120 Hz rectified signal (solid gray line), and the line‐filtered signals for 1L (solid purple), 3L (solid blue), and 5L (solid green), respectively. d) A magnified plot of voltage versus time for three samples to compare the leftover ripple voltages.

Since the M‐Fe_3_O_4_/Ni electrodes demonstrate excellent properties at 120 Hz, we evaluate the AC line‐filtering performance of the electrodes using the electric circuit shown in **Figure** **9**a. A sinusoidal voltage signal at 60 Hz is rectified by the bridge diode to the unidirectional signal at 120 Hz with the peak voltage of *V*_p_, as exhibited in Figure 9b. The rectified signal is then smoothened to a line‐filtered signal by the capacitor connected to the bridge diode. The line‐filtered signal is centered at the mean voltage of *V*_DC_ with the leftover ripple of *V*_r_ (Figure 9b). We show the results of our line‐filtering experiments in Figure 9c when using a symmetric two‐electrode supercapacitor consisting of two identical electrodes like 1L–1L, 3L–3L, or 5L–5L separated by the liquid electrolyte, as the capacitor in the electric circuit. The unidirectional voltage signal at 120 Hz rectified from an initial sinusoidal voltage signal at 60 Hz is successfully line‐filtered by the 1L‐, 3L‐, and 5L‐based symmetric supercapacitors; for all three cases, the filtered signals are nearly in a straight line and the remaining ripples are negligible. The reason for the increased *V*_DC_ with an increasing number of porous layers is because *V*_DC_ is equal to *V*_p_ − 0.5*V*_r_, and *V*_r_ decreases with increasing *C*_A._^[^ ^96^ ^]^ To assess *V*_r_ for three cases, the zoom‐in view of Figure 9c is exhibited in Figure 9d. *V*_r_, defined by the difference between the maximum and the minimum of a fluctuating signal, are 18.2, 12.2, and 12.1 mV for 1L, 3L, and 5L, respectively. This is in good agreement with the increasing trend of *C*_A_ with the number of porous layers. Note that even the largest ripple observed in the 1L case is similar to that in the commercial AEC, indicating the excellent of our electrodes as the capacitor for AC line‐filtering.^[^ 1, 118 ^]^

## 3 Conclusion

We propose a new approach to enabling ultrafast energy storage using 3D periodic porous supercapacitive Fe_3_O_4_/Ni electrodes that are hierarchically engineered across the three different length scales. The combination of: 1) the epitaxial Ni scaffolds for facile electron transport (atomic‐scale); 2) the ultrathin (≈5 nm) Fe_3_O_4_ nanosheets for maximized utilization of surface‐limited charge storage (nanoscale); 3) the periodically arranged 500 nm‐diameter pores for rapid ion diffusion (mesoscale) yields excellent response kinetics under both DC and AC signals. The ability of our approach to control the number of porous layers makes it more attractive as a versatile platform since it can freely manipulate the electrode properties suitable for the target application (e.g., 5L for DC and 1L for AC). We expect that electrode performance can be much enhanced by further optimization such as using epitaxial metal scaffolds with fewer defects via better lattice match, controlling the morphology or composition of electrochemically active oxides, and engineering the pore size. We believe that the concept of hierarchical multiscale engineering can extend its utility to many branches of applications including photoelectrochemistry, batteries, and electrocatalysts where an efficient electron and ion transport is highly desirable.

## 4 Experimental Section

4.1

4.1.1

##### Substrate Preparation

The *c*‐plane sapphire (Al_2_O_3_ (0001); I‐Nexus, Inc., Republic of Korea) substrates were used for epitaxial growth of 3D mesostructured Ni current collector (M‐Ni). The substrates were cleaned with piranha solution (3:1 H_2_SO_4_:H_2_O_2_ by volume) for an hour and rinsed with Type 1 water (18.2 MΩ cm^−1^). The substrates were dried with N_2_ gas flow. A 5 nm‐thick Ti layer followed by a 100 nm‐thick Au layer was deposited on the substrate using an electron beam evaporator (KVE‐E2000L Series, Korea Vacuum Tech, Republic of Korea).

##### Colloidal Crystal Growth

The Au/Ti/sapphire substrates were cut into 0.6 cm‐wide pieces, cleaned with piranha solution, and rinsed with Type 1 water. The substrates were immersed into an aqueous solution of 1 m sodium 3‐mercapto‐1‐propanesulfonate (90%, Sigma‐Aldrich, USA) overnight. The substrates were then placed vertically within vials filled with a 2% w/v aqueous suspension of 500 nm‐diameter PS beads (8% w/v sulfate latex, Thermo Fisher Scientific, USA) at 55 °C overnight and subsequently annealed at 95 °C for 5 h to promote the adhesion between PS spheres.

##### Electrodeposition of Ni and Fe_3_O_4_

To fabricate 3D mesostructured epitaxial Ni (M‐Ni), the procedure reported elsewhere was reported with minor modifications.^[^ ^122^ ^]^ Two‐electrode galvanostatic electrodeposition of Ni was conducted at a current density of −2.0 mA cm^−2^ in a Ni plating solution (Semi‐bright finish, Thermo Fisher Scientific, USA) with the colloidal crystals assembled on the Au/Ti/sapphire as a working electrode and a Ni plate as a counter electrode, respectively. The number of porous Ni layers was controlled by adjusting the electrodeposition time. The electrodeposited samples were immersed in THF (99.5%, JUNSEI, Japan) for 2–3 days to remove PS particles. As a control, a 100 nm‐thick dense Ni film (D‐Ni) was electrodeposited on the bare Au/Ti/sapphire.

Fe_3_O_4_ was electrodeposited in a three‐electrode configuration based on the procedure reported elsewhere.^[^ 53, 123 ^]^ M‐Ni or D‐Ni was used as a working electrode, a Pt plate as a counter electrode, and a 4 m KCl saturated Ag/AgCl electrode as a reference electrode. The electrolyte was an aqueous solution of 43.3 mm Fe_2_(SO_4_)_3_·*x*H_2_O (97%, Sigma‐Aldrich), 100 mm triethanolamine (99%, Sigma‐Aldrich), and 2 m NaOH (98%, Alfa Aesar) at 80 °C. The voltage profile applied was a repeated (200 times) sequence consisting of −1.04 V versus Ag/AgCl for 1 ms and open‐circuit voltage (OCV) for 10 s.

##### Characterization

The morphology of the sample was characterized by plan‐view and cross‐sectional SEM (Regulus 8230, Hitachi High‐Tech, Japan). The crystallography along the out‐of‐plane and in‐plane orientations was investigated by collecting 2*θ*/*ω* scans, rocking curves, and pole figures using XRD (AutoMATE II, Rigaku, Japan) with Cu K*α* radiation (λ = 0.15148 nm). The microstructure and crystal structure of the cross‐sectional sample prepared by FIB milling (Helios NanoLab 600, FEI, USA) were examined using TEM (Tecnai G2 F20, FEI, USA) with a 200 kV acceleration voltage. Elemental mappings were conducted using an EDX attached to the TEM. The vibrational modes were characterized using Raman spectroscopy (UniRAM, UniNano Tech, Republic of Korea) with a 532 nm excitation wavelength. The chemical states and nonstoichiometry were analyzed by XPS (Nexsa, Thermo Scientific, USA) with a monochromatic Al Kα source.

##### Electrochemical Measurement

CV, GCD, and EIS measurements were conducted on a three‐electrode setup at room temperature using a single‐channel potentiostat (SP‐200, Bio‐Logic, USA). The fabricated samples, a Hg/HgO electrode, and a Pt wire were used as a working electrode, a reference electrode, and a counter electrode, respectively. An aqueous solution of 6 m KOH (90%, Sigma‐Aldrich, USA) was used as an electrolyte. The CV and GCD measurements were performed over a potential window of −0.6 to 0.0 V. The EIS spectra were collected by applying a sinusoidal voltage amplitude of 10 mV at an equilibrium open‐circuit potential over the frequency ranging from 2 MHz to 0.1 Hz.

##### AC Line‐Filtering

An electric circuit comprising a four‐bridge‐diode rectifier connected to a capacitor in parallel with a load resistor (1 kΩ) is used for evaluating AC line‐filtering performance. The capacitor was the symmetric two‐electrode supercapacitor composed of the 6 m KOH aqueous electrolyte sandwiched between two identical Fe_3_O_4_/Ni electrodes such as 1L–1L, 3L–3L, or 5L–5L. A handheld oscilloscope (Analog Discovery 2, Digilent, USA) was used for generating the input AC signal and also collecting the output signal. The input signal with 1 V amplitude and 60 Hz frequency was applied to two nodes of the bridge diode. The output signal was acquired at the terminal of the load resistor in parallel with our two‐electrode supercapacitor that is connected to the other nodes of the bridge diode.

## Conflict of Interest

The authors declare no conflict of interest.

## Supporting information

Supplementary Material

## Data Availability

The data that support the findings of this study are available from the corresponding author upon reasonable request.
