# Supplementary material for: Hierarchical Multiscale Engineered Fe3O4/Ni Electrodes with Ultrafast Supercapacitive Energy Storage for Alternate Current Line‐Filtering
Source: Small Sci. 2022 Dec 14;3(2):2200074. doi: 10.1002/smsc.202200074 (PMC11935884; doi:10.1002/smsc.202200074)
Supplement: Supplementary file 1 — Supplementary Material [file SMSC-3-2200074-s001.pdf]

Supporting Information

Hierarchical Multiscale Engineered Fe_3O_4/Ni Electrodes with Ultrafast Supercapacitive Energy Storage for AC Line-Filtering

*Minjeong Kim, Byeong-Kwon Ju, Jin Gu Kang**

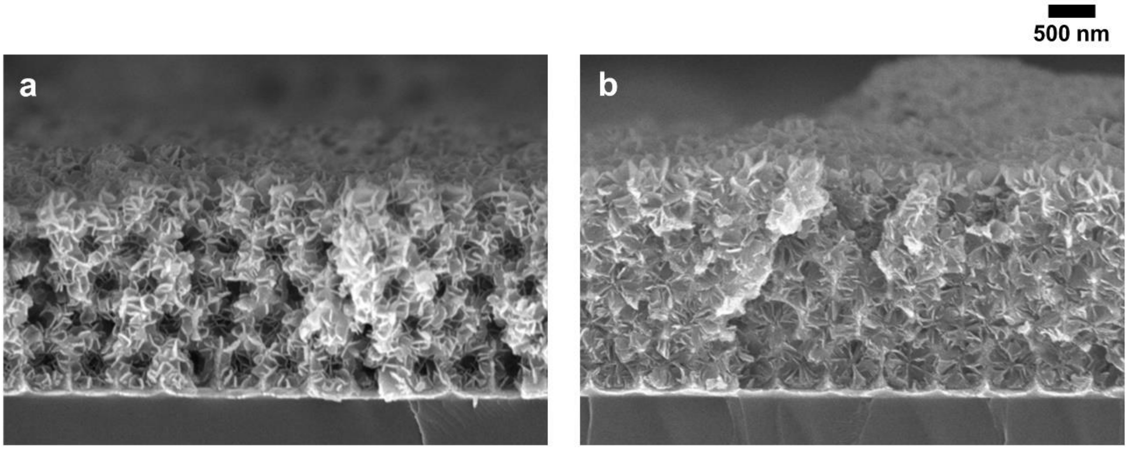

Figure S1. Cross-sectional SEM images of M-Fe_3O_4/Ni electrodes fabricated by Fe_3O_4 pulsed electrodeposition (ON: -1.04 V for 1 ms; OFF: open circuit potential for 10 s) with different cycle numbers: (a) 250 and (b) 500 cycles.

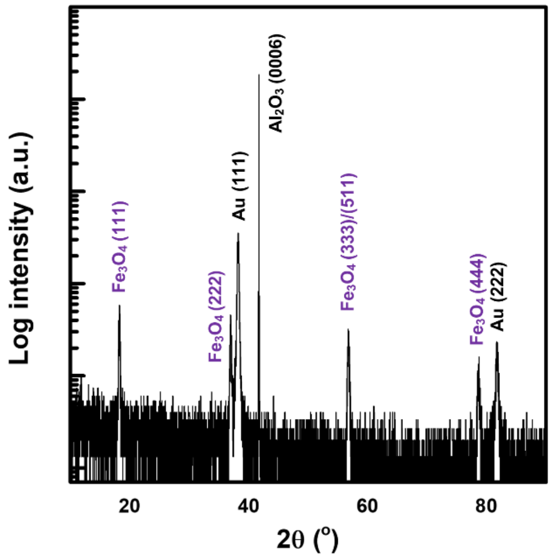

Figure S2. $2\theta/\omega$ scan of Fe_3O_4 film on Au/Ti/sapphire substrate fabricated by applying continuous potential (-1.04 V for 5 min).

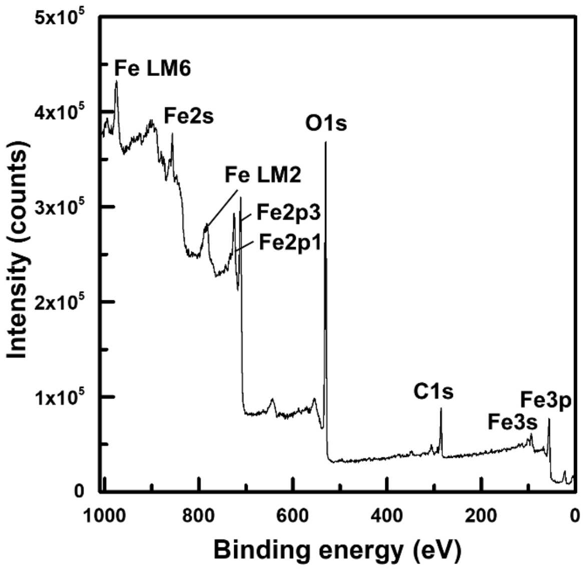

Figure S3. Survey XPS spectrum of M-Fe_3O_4/Ni.

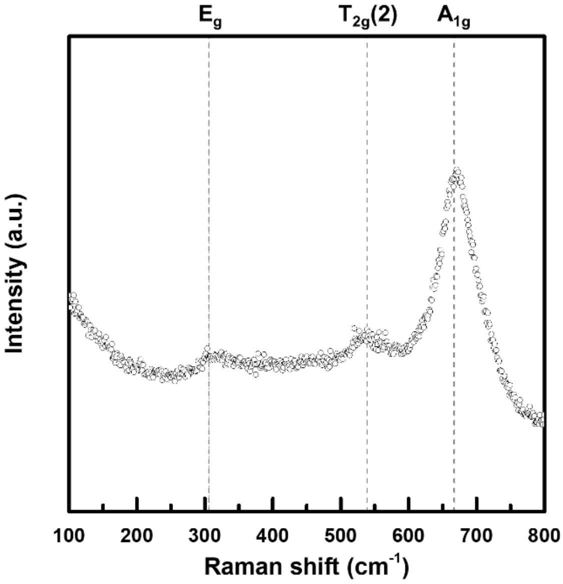

Figure S4. Raman spectrum of M-Fe_3O_4/Ni.

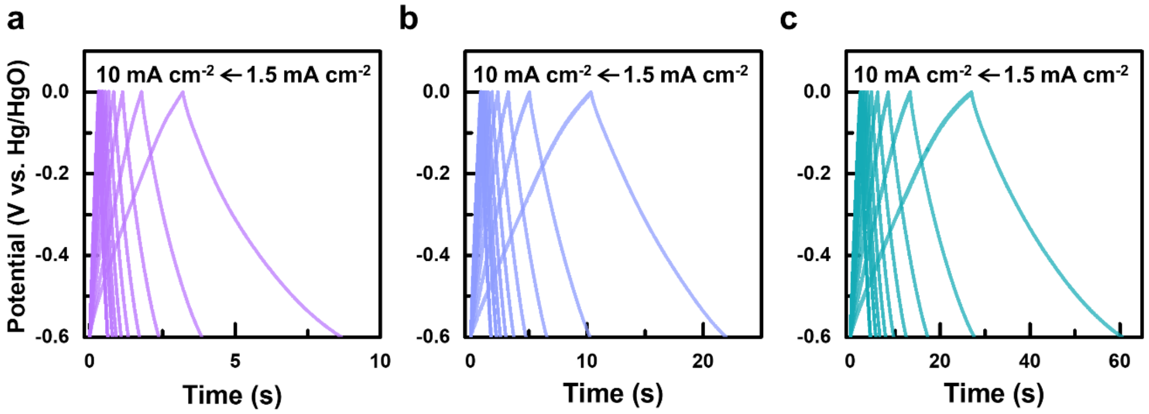

Figure S5. GCD profiles of (a) 1L, (b) 3L, and (c) 5L at current densities ranging from 1.5 mA cm^{-2} to 10 mA cm^{-2}.

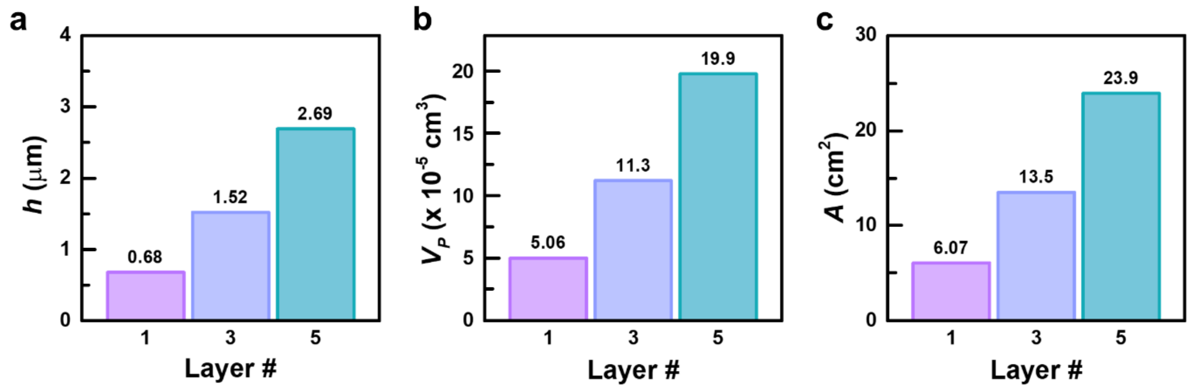

Figure S6. (a) Thickness (*h*) of the M-Fe_3O_4/Ni electrodes. (b) Total pore volume (V_P) of the electrodes calculated from FCC packing density. (c) Calculated surface area (*A*) of the electrodes.

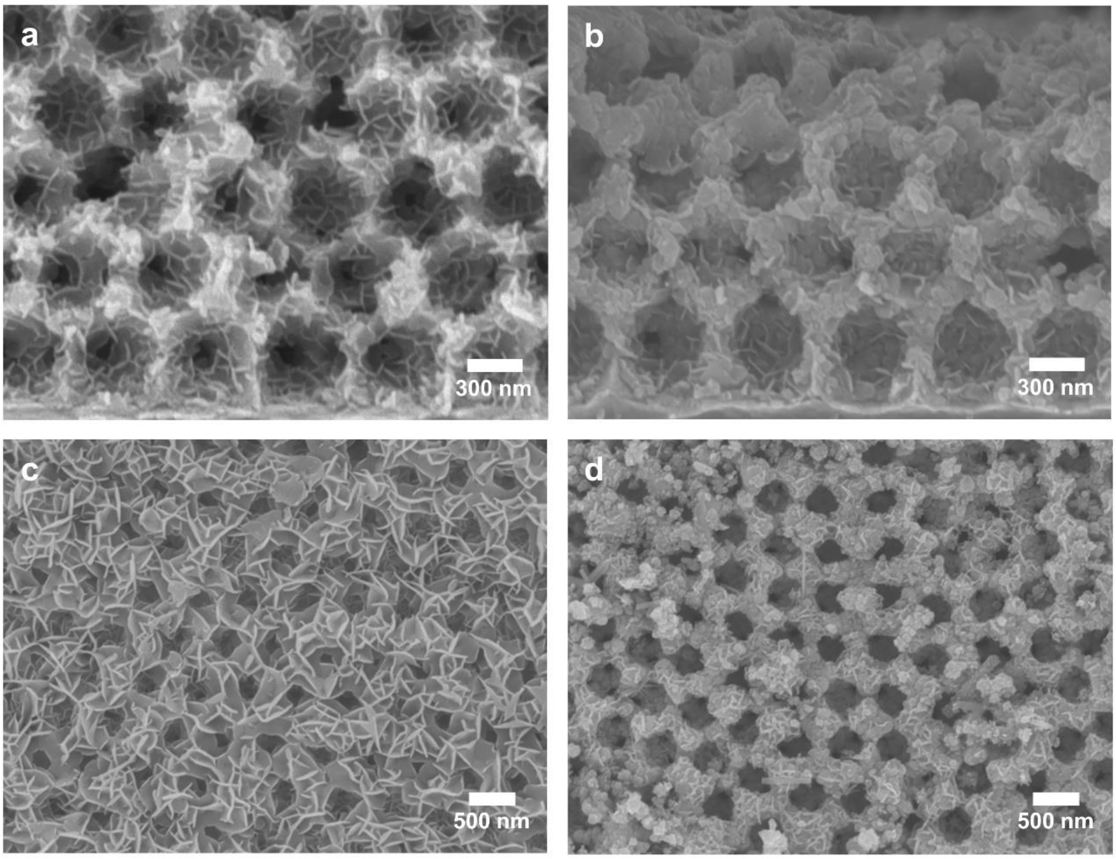

Figure S7. (a,b) Cross-sectional and (c,d) plan-view SEM images of M-Fe_3O_4/Ni before (a,c) and after (b,d) charge-discharge cycling.

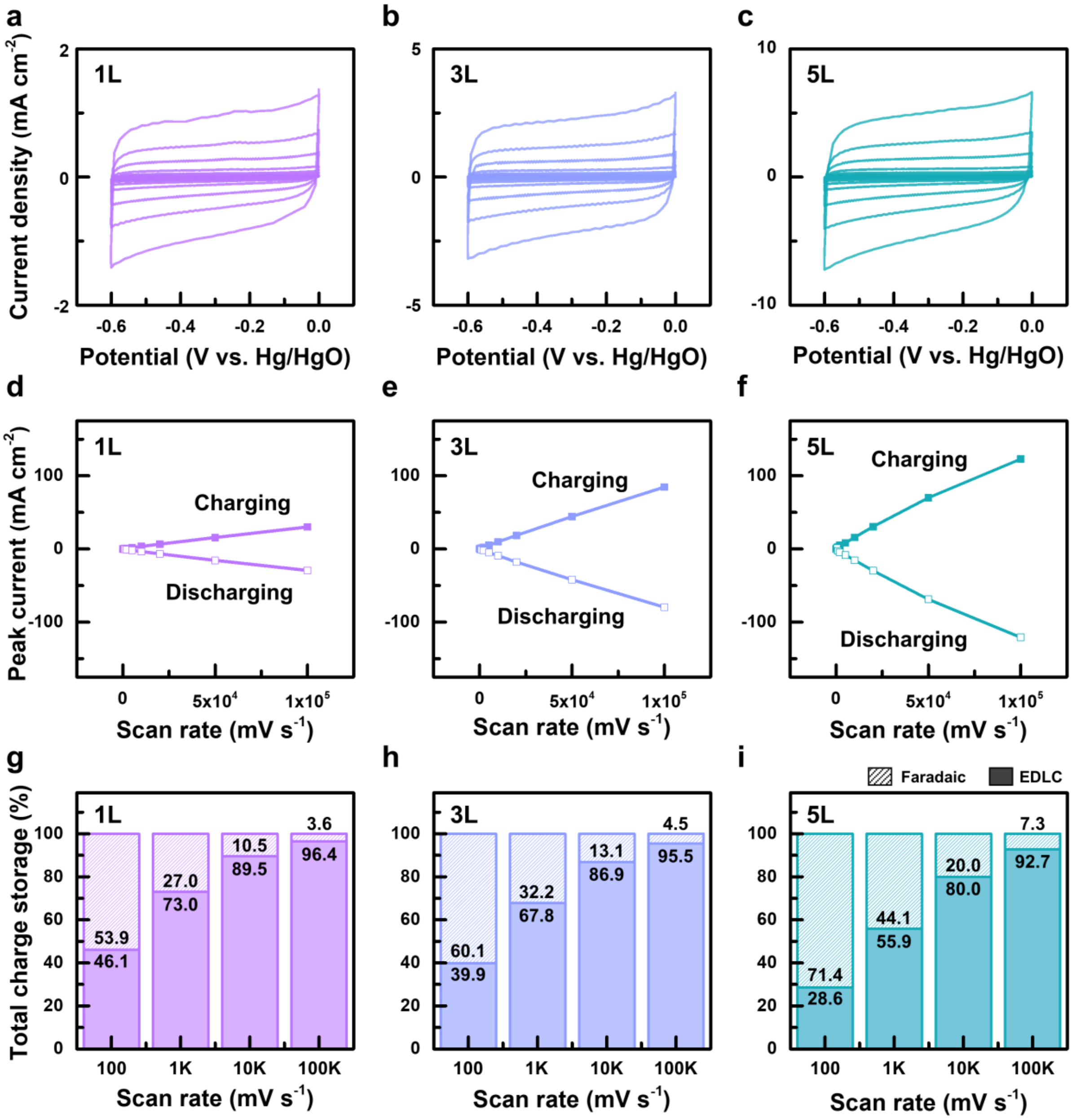

Figure S8. (a-c) CV curves for 1L (a), 3L (b), and 5L (c) at low scan rates over 10 mV s^{-1} - 2 V s^{-1}. (d-f) Plots of CV charge/discharge currents vs. scan rate for 1L (d), 3L (e), and 5L (f). (g-i) Relative contributions of EDLC and pseudocapacitance as a function of scan rate for 1L (g), 3L (h), and 5L (i).

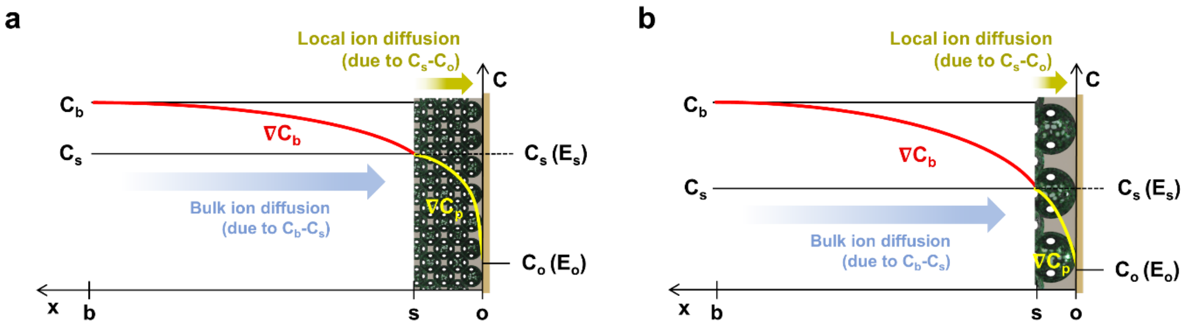

Figure S9. Schematic illustrations of chemical diffusion in (a) 5L and (b) 1L. *C* and *E* (< 0; cathodic) denote the concentration and the potential, respectively. The subscripts *b*, *s*, and *o* are the electrolyte bulk, the sample surface, and the Au metal surface (where the external cathodic potential is applied), respectively. The concentration gradients are drawn using solid curves to show the rate of diffusion.

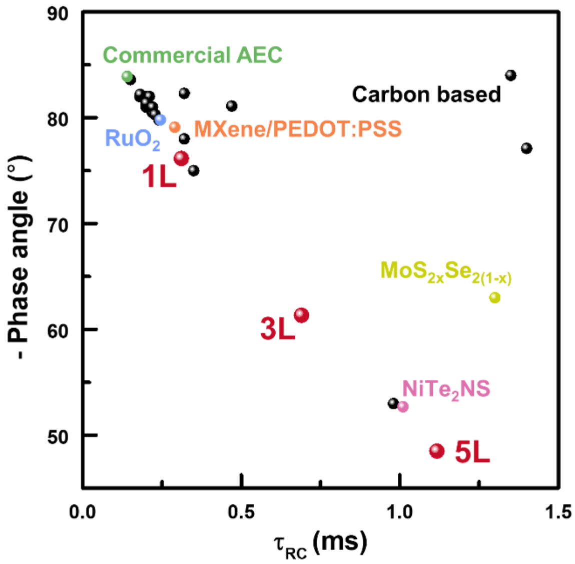

Figure S10. A summarized graph plotting phase angle vs. τ_{RC} for M-Fe_3O_4/Ni electrodes (red circles) and other previously reported electrodes for comparison.

Table S1. Performance summary of ultrafast supercapacitors for AC line filtering

Electrodes		At 120 Hz			ESR (Ω)	τ_0 (ms)
		τ_{RC} (ms)	C_A (μF cm^{-2})	-Phase (°)		
This work	1L	0.31	272	76.2	0.028	1.0
	3L	0.69	856	61.3	0.088	4.0
	5L	1.12	1688	48.5	0.126	7.9
PEDOT//ErGO[1]		0.18	270	82	0.21	0.46
ERLGO[2]		0.219	151	80.5	0.55	0.56
VOGN[3]		0.2	175	82	1.1	0.067
rGO fibers[4]		0.47	264	81.1	N/A	N/A
ErGO[5]		1.35	283	84	0.1	0.238
3OPG[6]		0.98	755	53	3.5	1.46
SWNT[7]		0.2	601	81	0.25	0.7
VOGN[8]		N/A	265	85	0.08	0.05
POG/NF[9]		0.21	360	82	0.07	0.248
C sponges[10]		0.319	487	78	N/A	0.37
C black[11]		0.35	559	75	0.44	1.56
NHG[12]		0.20	478	81.2	0.22	0.82
EOG/CCP[13]			600	83	0.079	0.083
GMF[14]		0.32	306	82.3	0.37	0.16
G/PEDOT:PSS[15]		0.2	543	81.4	0.147	0.57
VOGN[16]		N/A	80	86	0.6 - 0.7	1
G-V_2O_3/VO_x[17]		N/A	N/A	N/A	0.15	8.77
Ni/Ru/RuO_2[18]		0.245	515	79.8	N/A	0.69
$NiTe_2$ NS[19]		1.01	846	52.7	0.69	7.3
$MoS_{2x}Se_{2(1-x)}$[20]		1.3	448	63	N/A	0.7
$MoSe_2$ NS[21]		N/A	N/A	60	8.4	0.036
TiN NC[22]		N/A	N/A	41	1.4	2.5
AT-PEDOT:PSS[23]		0.15	994	83.6	0.09	0.588
SWCNT[24]		0.181	282	82.2	0.26	0.501
CMK-3/CNT[25]		0.228	559	80.3	0.25	1
NG-P[26]		1.4	318	77.1	N/A	0.05
G/PEDOT:PSS[27]		0.644	179	N/A	2.56	2.9
KB-CNT[28]		0.24	574	79.8	0.28	1
MXene/PEDOT:PSS[29]		0.29	560	79.1	0.29	0.709
Commercial AEC[30]		0.14	300	83.9	N/A	N/A

Equation S1.[31] $C = \frac{\int IdV}{2v\Delta VA}$

(C: areal capacitance calculated from CV, $\int IdV$: closed curve area, v: scan rate, ΔV: potential window, A: electrode area)

Equation S2.[32] $C_A = -\frac{1}{2\pi f Z'' A}$

(C_A: areal capacitance in response to AC input, f: frequency, Z'': imaginary part of impedance)

Equation S3.[33] $\theta = \tan^{-1}\left(\frac{Z''}{Z'}\right)$

(θ: phase angle, Z': real part of impedance)

Equation S4.[32] $\tau_{RC} = -\frac{Z'}{2\pi f Z''}$

(τ_{RC}: resistor-capacitor time constant)

Equation S5.[32, 34] $C'' = \frac{Z'}{2\pi f |Z|^2}$

(C'': complex capacitance, |Z|: impedance magnitude)

Equation S6.[32, 35, 36] $k_w = \frac{RT}{n^2 F^2 A\sqrt{2}}\left(\frac{1}{D_{ion}{}^{1/2} C^*}\right)$

(k_w: Warburg coefficient, R: gas constant, T: absolute temperature, n: number of electrons transferred, F: Faraday constant, D_{ion}: diffusion coefficient, C^*: ion concentration)

Supplementary Note S1. Based on the previous report[37] and the universal feature of transition metal oxides (i.e., multiple possible oxidation states like M^{2+} and M^{3+}), we propose the following surface-redox reaction as the likely origin of Fe_3O_4 pseudocapacitance:

$Fe_3O_4 + OH^- + H_2O + e^- \leftrightarrow 3FeOOH$

During charge, the surface of Fe_3O_4 reacts with OH^- and H_2O in the electrolyte to form FeOOH. During discharge, FeOOH transforms to Fe_3O_4 and releases OH^- and H_2O. This process is not much different from those of many pseudocapacitive transition metal oxides. Taking MnO_2 as an example[38-39], it adopts $MnO_2 + H_2O + e^- \leftrightarrow MnOOH + OH^-$. The reason for co-existence of OH^- and H_2O at the reactant side for Fe_3O_4 is because Fe_3O_4 contains mixed oxidation states of Fe^{2+} and Fe^{3+} whereas MnO_2 contains Mn^{4+} only.

Supplementary Note S2. We calculate the surface areas of a series of our samples using the following argument. Since the amount of Fe_3O_4 is significantly smaller relative to Ni, it would be reasonable to evaluate the surface areas of the Ni scaffolds only. As the first step, we measured the thickness of each sample using the cross-sectional SEM image and plotted them in **Figure S6**a. Since the geometric area of all samples were controlled to be 1 cm^2 and the packing density of self-assembled FCC-type opal templates is typically 74%, the total pore volumes (V_P) in 1L, 3L, and 5L are calculated *via* the following equation,

$$V_P = 0.74 \times h \times A_g$$

where h and A_g are the measured thickness and the geometric area, respectively (**Figure S6**b). As the volume and the surface area of one pore are $4\pi r^3/3$ and $4\pi r^2$ (r = 250 nm in our case), respectively, the surface area of all pores inside (A), which is identical to the surface area of the Ni scaffold, can be extracted as follows:

$$A = \frac{3V_P}{4\pi r^3} \times 4\pi r^2 = \frac{3V_P}{r}.$$

Figure S6c shows the plot of the surface area against the number of porous layers. The increasing trend of the surface area from 1L, 3L to 5L is clearly observed.

Supplementary Note S3. The capacitance of pseudocapacitive materials is the sum of the capacitances contributed by surface-controlled non-faradaic and diffusion-controlled faradaic processes. The ratio between the two processes is determined by analyzing the dependence of the mean current density (i) on the scan rate (v): $i = i_s + i_d = k_1 v + k_2 v^{1/2}$ [40-42], where i_s (= $k_1 v$) and i_d (= $k_2 v^{1/2}$) denote the current densities of non-faradaic EDLC and faradaic redox processes, respectively.

Supplementary Note S4. We interpret our observation on faster diffusion in 5L relative to 1L based on the following statement. We know that what we prove in the experiment is the chemical diffusion process, which is dependent on the ion concentration gradient (∇C); the larger ∇C, the faster diffusion is. **Figure S9** illustrates the potential profiles and the resulting concentration profiles created in our electrochemical cells under a cathodic potential applied (E_o). The magnitude of the potential at the sample surface (E_s), is reduced by iR, where i and R are the steady-state current and the sample resistance, respectively. Typical dense conductive samples have negligible R, yielding $E_o = E_s$. However, porous samples exhibit the large R, leading to $E_o < E_s$. Since R of 5L is much higher than that of 1L, E_s of 5L is higher (i.e., less negative) relative to 1L. As a result, 5L exhibits the higher ion concentration at the surface (C_s) than 1L.

In this scenario, ∇C can be divided into two parts: one is the ion concentration gradient over the bulk diffusion layer (∇C_b) and the other is over the porous sample layer (∇C_p). As shown in **Figure S9**, ∇C_b for 1L is larger relative to 5L because the ion concentration difference (ΔC_b) between the bulk electrolyte (C_b) and the sample surface (C_s) over the ~100 μm-thick typical Nernst diffusion layer for 1L is larger than that for 5L. In contrast, ∇C_p for 5L is larger relative to 1L since ΔC_p between the sample surface (C_s) and the metal surface (C_o) over the porous layer for 5L is larger than that for 1L. Note that although the thickness of the porous layer in 1L is a factor of ~5 smaller than that in 5L, ∇C_p for 5L can still be much larger relative to 1L because the ion concentration varies exponentially with the potential based on the Nernstial equation as follows:

$$\Delta E = E_2 - E_1 = \frac{RT}{F} \ln \frac{C_2}{C_1}$$

$$C_2 = C_1 e^{\frac{F\Delta E}{RT}} = C_1 e^{\frac{\Delta E}{0.0256}}$$

where F, R, and T are the Faraday constant, gas constant, and absolute temperature. At room temperature, RT/F = 25.6 mV.

For ∇C_b, chemical diffusion in 1L is faster than 5L whereas for ∇C_p, it is vice versa. The final step is therefore to determine which ion concentration gradient has a stronger effect on the entire diffusion process: ∇C_b vs. ∇C_p. As the ion concentration varies exponentially for both cases, the critical factor is the thickness of the layer where chemical diffusion occurs. The thickness of bulk diffusion layer is typically on the order of ~100 μm. In contrast, the thicknesses of the porous layer are ~0.5 and ~2 μm for 1L and 5L, respectively, which are 2 –

3 orders of magnitude smaller relative to bulk diffusion layer. Therefore, it is reasonable to conclude that ∇C_p is the most critical, and hence that chemical diffusion in 5L can be much faster than 1L.

References

[1] M. M. Wu, F. Y. Chi, H. Y. Geng, H. Y. Ma, M. Zhang, T. T. Gao, C. Li, L. T. Qu, *Nat. Commun.* **2019**, 10, 2855.

[2] F. Y. Chi, C. Li, Q. Q. Zhou, M. Zhang, J. Chen, X. W. Yu, G. Q. Shi, *Adv. Energy Mater.* **2017**, 7, 1700591.

[3] J. R. Miller, R. A. Outlaw, B. C. Holloway, *Science* **2010**, 329, 1637.

[4] J. X. Zhao, Y. Zhang, J. X. Yan, X. X. Zhao, J. X. Xie, X. Luo, J. H. Peng, J. J. Wang, L. C. Meng, Z. M. Zeng, C. H. Lu, X. H. Xu, Y. F. Dai, Y. G. Yao, *ACS Appl. Energy Mater.* **2019**, 2, 993.

[5] K. X. Sheng, Y. Q. Sun, C. Li, W. J. Yuan, G. Q. Shi, *Sci. Rep.* **2012**, 2, 247.

[6] J. L. Xue, Z. S. Gao, L. Y. Xiao, T. T. Zuo, J. Gao, D. W. Li, L. T. Qu, *ACS Appl. Energy Mater.* **2020**, 3, 5182.

[7] Y. Rangom, X. Tang, L. F. Nazar, *ACS Nano* **2015**, 9, 7248.

[8] M. Z. Cai, R. A. Outlaw, R. A. Quinlan, D. Premathilake, S. M. Butler, J. R. Miller, *ACS Nano* **2014**, 8, 5873.

[9] G. F. Ren, X. Pan, S. Bayne, Z. Y. Fan, *Carbon* **2014**, 71, 94.

[10] J. Joseph, A. Paravannoor, S. V. Nair, Z. J. Han, K. Ostrikov, A. Balakrishnan, *J. Mater. Chem. A* **2015**, 3, 14105.

[11] P. Kossyrev, *J. Power Sources* **2012**, 201, 347.

[12] Q. Q. Zhou, M. Zhang, J. Chen, J. D. Hong, G. Q. Shi, *ACS Appl. Mater. Interfaces* **2016**, 8, 20741.

[13] G. F. Ren, S. Q. Li, Z. X. Fan, M. N. F. Hogue, Z. Y. Fan, *J. Power Sources* **2016**, 325, 152.

[14] Z. Y. Zhang, M. L. Liu, X. Tian, P. Xu, C. Y. Fu, S. Wang, Y. Q. Liu, *Nano Energy* **2018**, 50, 182.

[15] M. Zhang, X. W. Yu, H. Y. Ma, W. C. Du, L. T. Qu, C. Li, G. Q. Shi, *Energy Environ. Sci.* **2018**, 11, 559.

[16] D. Premathilake, R. A. Outlaw, S. G. Parler, S. M. Butler, J. R. Miller, *Carbon* **2017**, 111, 231.

[17] X. Pan, G. F. Ren, M. N. F. Hoque, S. Bayne, K. Zhu, Z. Y. Fan, *Adv. Mater. Interfaces* **2014**, 1, 1400398.

[18] A. Morag, N. Maman, N. Froumin, V. Ezersky, K. Rechav, R. Jelinek, *Adv. Electron. Mater.* **2020**, 6, 1900844.

[19] H. C. Tang, K. Q. Xia, J. G. Lu, J. M. Fu, Z. Y. Zhu, Y. Tian, Y. Wang, M. Q. Liu, J. Chen, Z. W. Xu, Y. C. Guo, R. Khatoon, H. W. Chen, Z. Z. Ye, *Nano Energy* **2021**, 84, 105931.

[20] A. Sellam, R. N. Jenjeti, S. Sampath, *J. Phys. Chem. C* **2018**, 122, 14186.

[21] Z. Jiang, Y. Wang, S. G. Yuan, L. Shi, N. Wang, J. Xiong, W. H. Lai, X. Y. Wang, F. Y. Kang, W. Lin, C. P. Wong, C. Yang, *Adv. Funct. Mater.* **2019**, 29, 1807116.

[22] P. H. Yang, D. L. Chao, C. R. Zhu, X. H. Xia, Y. Q. Zhang, X. L. Wang, P. Sun, B. K. Tay, Z. X. Shen, W. J. Mai, H. J. Fan, *Adv. Sci.* **2016**, 3, 1500299.

[23] M. Zhang, Q. Q. Zhou, J. Chen, X. W. Yu, L. Huang, Y. R. Li, C. Li, G. Q. Shi, *Energy Environ. Sci.* **2016**, 9, 2005.

[24] Y. Yoo, S. Kim, B. Kim, W. Kim, *J. Mater. Chem. A* **2015**, 3, 11801.

[25] Y. Yoo, M. S. Kim, J. K. Kim, Y. S. Kim, W. Kim, *J. Mater. Chem. A* **2016**, 4, 5062.

[26] Y. Z. Chang, G. Y. Han, D. Y. Fu, F. F. Liu, M. Y. Li, Y. P. Li, C. X. Liu, *Electrochim. Acta* **2014**, 115, 461.

[27] Z. S. Wu, Z. Y. Liu, K. Parvez, X. L. Feng, K. Mullen, *Adv. Mater.* **2015**, 27, 3669.

[28] Y. Yoo, J. Park, M. S. Kim, W. Kim, *J. Power Sources* **2017**, 360, 383.

[29] G. S. Gund, J. H. Park, R. Harpalsinh, M. Kota, J. H. Shin, T. I. Kim, Y. Gogotsi, H. S. Park, *Joule* **2019**, 3, 164.

[30] K. S. Hwang, T. H. Yoon, C. W. Lee, Y. S. Son, J. K. Hwang, *J. Power Sources* **1998**, 75, 13.

[31] A. Allagui, T. J. Freeborn, A. S. Elwakil, B. J. Maundy, *Sci. Rep.* **2016**, 6, 38568.

[32] J. G. Kang, M. Kim, P. D. Kim, B. K. Ju, S. K. Kim, *Int. J. Energy Res.* **2022**, 46, 3490.

[33] M. G. Hosseini, E. Shahryari, *J. Solid State Electrochem.* **2017**, 21, 2833.

[34] E. M. Mills, S. Kim, *J. Electrochem. Soc.* **2020**, 167, 130506.

[35] T. Q. Nguyen, C. Breitkopf, *J. Electrochem. Soc.* **2018**, 165, E826.

[36] J. Zhang, H. J. Feng, Q. Qin, G. F. Zhang, Y. X. Cui, Z. Z. Chai, W. J. Zheng, *J. Mater. Chem. A* **2016**, 4, 6357.

[37] J. W. Halley, A. Schofield, B. Berntson, *J. Appl. Phys.* **2012**, 111, 124911.

[38] A. Kozawa, R. A. Powers, *J. Electrochem. Soc.* **1966**, 113, 870.

[39] J. Shin, J. K. Seo, R. Yaylian, A. Huang, Y. S. Meng, *Inter. Mater. Rev.* **2019**, 65, 356.

[40] N. R. Chodankar, H. D. Pham, A. K. Nanjundan, J. F. S. Fernando, K. Jayaramulu, D. Golberg, Y. K. Han, D. P. Dubal, *Small* **2020**, 16, 2002806.

[41] J. L. Liu, J. Wang, C. H. Xu, H. Jiang, C. Z. Li, L. L. Zhang, J. Y. Lin, Z. X. Shen, *Adv. Sci.* **2018**, 5, 1700322.

[42] V. Augustyn, P. Simon, B. Dunn, *Energy Environ. Sci.* **2014**, 7, 1597.
